# Platinum iodido drugs show potential anti-tumor activity, affecting cancer cell metabolism and inducing ROS and senescence in gastrointestinal cancer cells

**DOI:** 10.1038/s42003-024-06052-5

**Published:** 2024-03-22

**Authors:** Jorge Melones-Herrero, Sonia Alcalá, Laura Ruiz-Cañas, Carlos Benítez-Buelga, Sandra Batres-Ramos, Carmela Calés, Oscar Lorenzo, Rosario Perona, Adoración G. Quiroga, Bruno Sainz, Isabel Sánchez-Pérez

**Affiliations:** 1 Department of Biochemistry. School of Medicine, Universidad Autónoma de Madrid (UAM), Madrid, Spain; 2 Instituto de Investigaciones Biomédicas “Sols-Morreale” IIBM-CSIC-UAM, Madrid, Spain; 3 Biomarkers and Personalized Approach to Cancer (BioPAC) Group. Area 3 Cancer -Instituto Ramón y Cajal de Investigación Sanitaria (IRYCIS), Madrid, Spain; 4 Laboratory of Diabetes and Vascular Pathology, Instituto de Investigaciones Sanitarias-Fundación Jimenez Díaz, CIBERDEM, UAM, Madrid, Spain; 5 Centro de Investigación Biomédica en Red, Área Rare Diseases, CIBERER, ISCIII, Madrid, Spain; 6 Instituto de Salud Carlos III, Madrid, Spain; 7 Department of Inorganic Chemistry, School of Sciences, IAdChem, UAM, Madrid, Spain; 8 Centro de Investigación Biomédica en Red, Área Cáncer, CIBERONC, ISCIII, Madrid, Spain; 9 Unidad Asociada de Biomedicina UCLM-CSIC, Madrid, Spain

**Keywords:** Mechanism of action, Target identification

## Abstract

Cisplatin-based chemotherapy has associated clinical disadvantages, such as high toxicity and resistance. Thus, the development of new antitumor metallodrugs able to overcome different clinical barriers is a public healthcare priority. Here, we studied the mechanism of action of the isomers *trans* and *cis*-[PtI_2_(isopropylamine)_2_] (I5 and I6, respectively) against gastrointestinal cancer cells. We demonstrate that I5 and I6 modulate mitochondrial metabolism, decreasing OXPHOS activity and negatively affecting ATP-linked oxygen consumption rate. Consequently, I5 and I6 generated Reactive Oxygen Species (ROS), provoking oxidative damage and eventually the induction of senescence. Thus, herein we propose a loop with three interconnected processes modulated by these iodido agents: (i) mitochondrial dysfunction and metabolic disruptions; (ii) ROS generation and oxidative damage; and (iii) cellular senescence. Functionally, I5 reduces cancer cell clonogenicity and tumor growth in a pancreatic xenograft model without systemic toxicity, highlighting a potential anticancer complex that warrants additional pre-clinical studies.

## Abstract (web summary)

The study of the biomolecular mechanism of action of the isomers trans and cis-[PtI2(ipa)2] (I5 and I6) shows metabolic disruption, ROS generation and senescence. Functionally, I5 reduces tumor growth in vivo without systemic toxicity.

## Introduction

The development of metallocomplexes with potential applications in cancer treatment has been a hot topic since the approval, in 1978, of the anticancer drug cisplatin (CDDP). Despite the efficacy of platinum-based chemotherapy for the treatment of certain tumors, only a very limited number of new metallodrugs have gained clinical approval over the past 40 years. Nonetheless, research in this field has^1^ and continues^2,3^ to advance with new design approaches to improve pharmacological outcomes and, at the same time, reduce side-effects^4^.

Our group has expertize in designing antitumor platinum(II) complexes with non-conventional structures^5–7^. One approach in their design consists in modifying the leaving halide group, from chloride (in CDDP) to iodide. Iodido complexes have been excluded from anticancer drug libraries because in the earlier stages of CDDP development they were reported to be inactive^8^. Only a few publications late in the 1990s reconsidered the study of iodido complexes^9,10^, reporting some interesting clues about their solution behavior. Years later, our group took on the challenge of evaluating *cis* and *trans* Pt isomers bearing aliphatic amines as antitumor complexes, highlighting these prototypes as emerging therapeutics for the treatment of solid tumors with poor prognosis and for those cancers where conventional chemotherapies are ineffective^11–13^. Since then, a review of these platinum iodido drugs showed a common and interesting trend: some of these complexes are able to overcome CDDP resistance in different cancer cells^14^.

We have focused on the study of the biological effect of two iodido prototypes: the *trans* and *cis*-[PtI_2_(ipa: isopropylamine)_2_], hereafter I5 and I6, respectively. We found that both drugs induce apoptosis, partially independent of p53 (tumor suppressor protein), in contrast to CDDP^15^. These results increased the interest in these drugs for tumors that have p53 mutated (nearly 50% of solid tumors)^16^, highlighting other targets beyond DNA.

The mitochondria (i.e., metabolism) has emerged as a main target for the newest metallodrug designs due to its role in tumorigenesis and cell death processes^17,18^. However, although different metal complexes can produce mitochondrial damage, this is not always enough to achieve a therapeutic effect owing to the ability of tumor cells to alter their metabolic phenotypes for energy compensation (i.e., oxidative phosphorylation (OXPHOS) to glycolysis) in response to metabolic inhibitors^19,20^. Mitochondrial dysfunction can also activate cell death signaling pathways, including apoptosis, autophagy (and mitophagy) and senescence^21^. The role of senescence in cancer treatment is controversial, due to the dual action of the Senescence-Associated Secretory Phenotype (SASP)^22,23^; however, there is recent evidence showing the advantages of using a combinatorial therapy-based approach, where the first treatment is a chemotherapy that induces senescence in cancer cells followed by a second treatment with a senolytic that selectively kills these senescent cells^24^.

In addition to DNA adducts induced by CDDP, it is known that CDDP also produces oxidative stress, increasing reactive oxygen species (ROS)^25^ and provoking subsequent oxidative DNA damage^26^. ROS generation is a phenotype shared with other CDDP-derivatives and metallodrugs^27^. As a protective mechanism against the deleterious effects of excessive ROS production, cells express several enzymatic antioxidant defenses, such as superoxide dismutases, glutathione (GSH) peroxidase, and catalase, localized in distinct cellular compartments. Superoxide Dismutases (SODs) are a group of enzymes that selectively eliminate O_2_^·-^ forming hydrogen peroxide. SOD1 (Cu-Zn) can be found in the cytoplasm and in the mitochondrial intermembrane space; SOD2, also known as MnSOD, is responsible for converting mitochondrial O_2_^·-^^28^; and catalase, CAT, is likely the main enzyme to reduce H_2_O_2_ levels^29^.

Gastrointestinal (GI) cancer includes cancers in organs of the GI tract (e.g., esophageal, stomach, intestine, pancreas, colon, liver, rectum, anus, and biliary system), making GI cancer one of the most lethal diseases worldwide. Stomach (Gastric) cancer (GC) is the sixth most common diagnosed cancer and the third leading cause of cancer-related death^30,31^. The main treatment for this disease in its early stages is surgery and the administration of adjuvant radio-chemotherapy. The poor prognosis in GC patients is related to its late diagnosis and the development of cellular resistance to treatment^32^. Pancreatic Ductal Adenocarcinoma (PDAC) is the fourth most frequent cause of cancer-related death worldwide, primarily due to the inherent chemo-resistant nature and metastatic capacity of this tumor^33^. PDAC is highly resistant to standard of care chemotherapy and radiation, and due to a lack of early symptoms, patients are generally diagnosed with late advanced disease, characterized by extensive metastasis to secondary organs. Therefore, the clinical reality and the main problem associated with PDAC is an inability to effectively treat the disease at the time of diagnosis, highlighting the urgent healthcare need to develop more effective treatments for patients with advanced PDAC^34^.

In this manuscript, we aimed to study the mechanisms by which I5 and I6 affect two types of GI tumor cells in vitro. Towards this end, we measured cytotoxicity and evaluated DNA oxidation damage in GC and PDAC cell lines. We further studied the mitochondrial status and metabolism (OXPHOS and glycolysis) as well as cellular senescence after treatment and evaluated ROS generation and its possible role in cell viability and the aforementioned processes. We concluded that I5 and I6 induce oxidative DNA damage, senescence, increase ROS levels and we demonstrated that cell death may be related to these processes. Finally, we show that administration of I5 in vivo has potent anti-tumor effects in a PDAC cell xenograft model, with no apparent adverse effects on energy balance, nor on hematocrit, blood plasma or urine parameters. In summary, these data suggest that I5 and I6 represent new and safe iodine-based metal complexes that can be used to treat GI tumor cells, including GC and PDAC, warranting additional pre-clinical studies.

## Results

### The iodido prototypes cause DNA oxidative damage and mitochondrial dysfunction

**Fig. 1:**
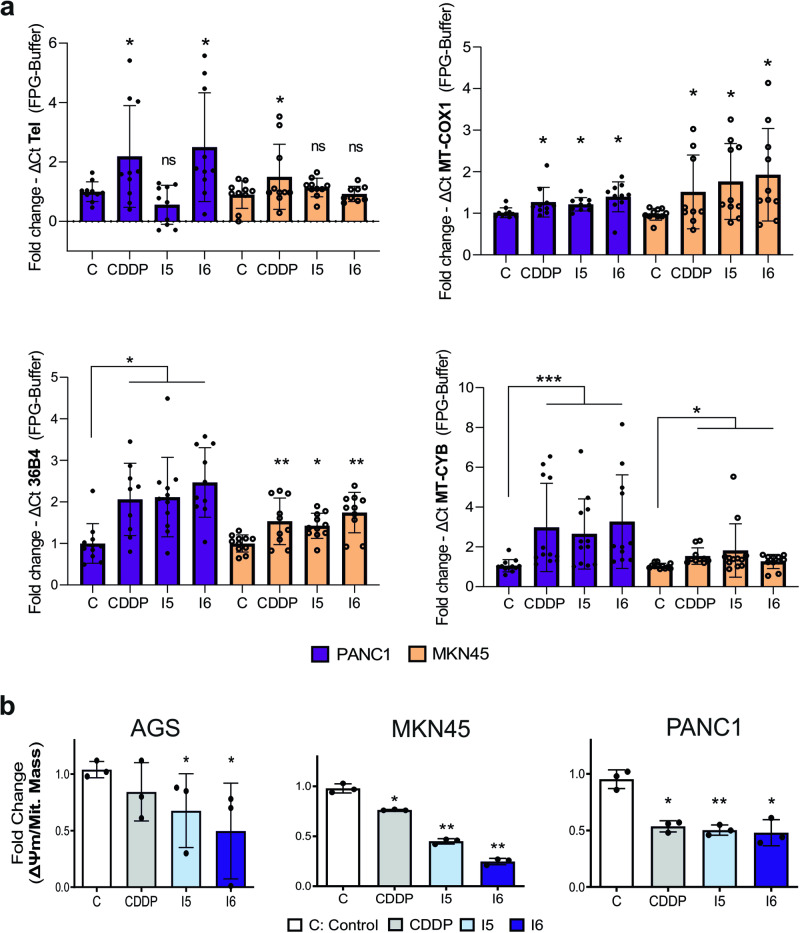
Iodido agents induce oxidative DNA damage and produce mitochondrial dysfunction in GI cancer cells. **a** Oxidative DNA damage (FPG sensitive lesions) at telomeres (left up), mitochondria regions encoding for mitochondrial MT-COX1 (right up) and MT-CYB (right down) genes, as well as the nuclear 36B4 single copy gene (left down) in cells exposed, or not, to IC_50_ concentrations (see Methods for details) of CDDP or compounds I5 or I6 during 24 h. The differences in PCR kinetics (ΔCt) between FPG-digested vs undigested DNA (Buffer) is represented for each sample. Bars represent the mean fold change ± SEM 10–12 replicates from three independent experiments normalized to the control. Statistical significance was calculated using two-tailed unpaired *t*-test (*p < 0.05, **p < 0.01, *** *p* < 0.001, ns not significant). **b** Mean fold change ± SD of the ratio of ΔΨm probe (CMX-ROS)/Mitochondrial mass probe (MitoGreen) as a measurement to evaluate mitochondrial functionality in AGS, MKN45 and PANC1 treated with CDDP, I5 or I6 (IC_50_ doses for 24 h). *p < 0.05, **p < 0.01, as determined by unpaired two-sided Student’s *t*-test, compared to untreated (C: Control) set as 1.0.

CDDP produces covalent binding, but the mechanism of action of platinum drugs is not simple and they also cause oxidative DNA damage^35^. Thus, we investigated potential DNA oxidation damage in the GC cell line MKN45 and the PDAC cell line PANC1 by qPCR amplification of three different target regions: telomeric (Tel), genomic (36B4) and mitochondrial (mtDNA: COX1 and CYB) following treatment with I5 and I6, as well as CDDP as a positive control. The results showed an increase in oxidative damage in all genes (except for Tel, Fig. 1a). As the mtDNA appeared to be the most affected and mtDNA damage is one of the main drivers of mitochondrial dysfunction, we measured mitochondrial mass and mitochondrial membrane potential (ΔΨm) in AGS (i.e., GC cell line), MKN45 and PANC1 cells continuously exposed to CDDP, I5 and I6 at their IC_50_ dose for 24 h. Mitochondrial mass and ΔΨ_m_ were measured by using MitoTracker™ Green and MitoTracker™ Red CMXRos, respectively (Fig. S1a) and used to calculate the ΔΨ_m_/Mit. Mass ratio (Fig. 1b), a way to normalize the membrane potential respective to the total mitochondrial mass (MM). Our results indicate that CDDP, I5 and I6 decrease the ΔΨ_m_/Mit. Mass ratio indicative of dysfunctional/damaged mitochondria.

### I5 and I6 modulate cell metabolism

**Fig. 2:**
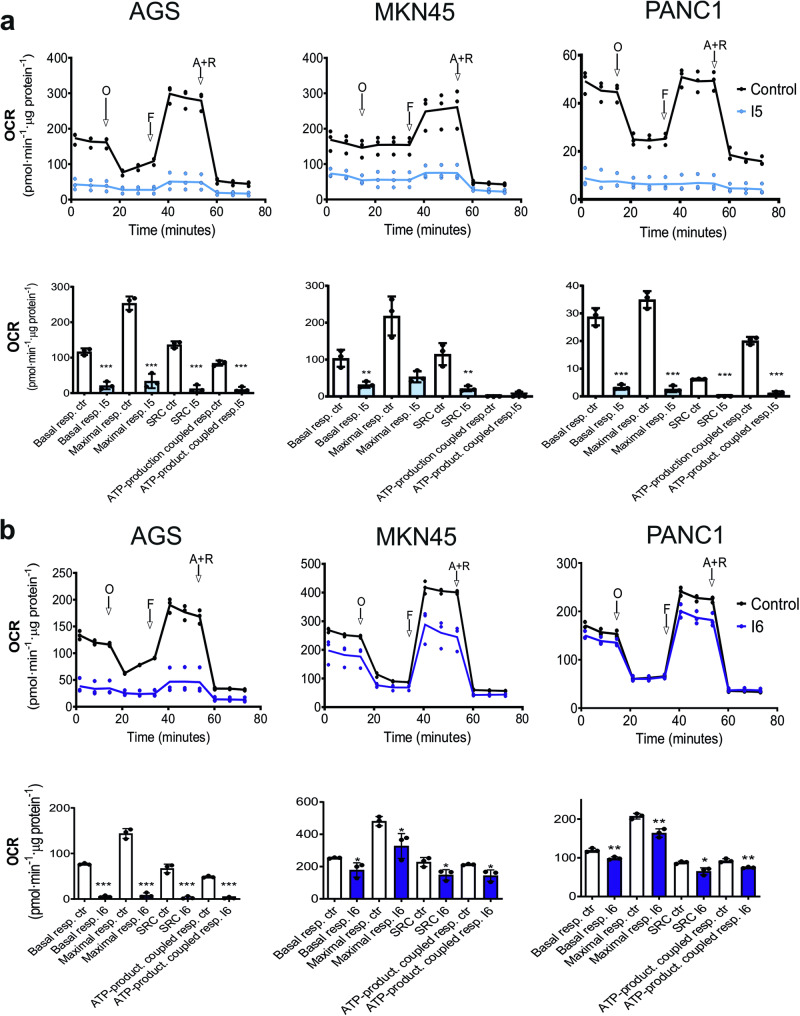
I5 and I6 affect gastric and pancreatic tumor cells oxygen consumption and mitochondrial functional properties. **a**, **b** (upper panels) Representative plots showing mean ± SD of the oxygen consumption rate (OCR) calculated for untreated (Control) and I5- or I6-treated AGS, MKN45 and PANC1 cells (according to IC_50_, 24 h), normalized to total protein using a BCA kit (measured as BCA absorbance). Cells were treated with distinct inhibitors of mitochondrial function: O (oligomycin), F (FCCP), A (antimycin A), and R (rotenone). Continuous OCR values (pmoles/min/µg protein) are shown. **a**, **b** (bottom panels) Mean ± SD of measured and calculated mitochondrial function parameters (*n* = 3 biological replicates with 3 readings). *p < 0.05, **p < 0.01, ***p < 0.001, as determined by unpaired two-sided Student’s *t*-test.

Mitochondria are the main cellular organelles responsible for energy production (i.e., ATP), and loss of ΔΨm results in ATP depletion, which is also associated with a metabolic switch from OXPHOS to glycolysis^36^. To investigate if I5 and I6 affect mitochondrial-mediated respiration in AGS, MKN45 and PANC1 cells, we measured the oxygen consumption rate (OCR) and extracellular acidification rate (ECAR) in cells as indicators of mitochondrial respiration and glycolysis, respectively (Fig.2a-b, S1b-c). Specifically, using the Seahorse XF extracellular flux analyzer, OCR in cells 24 h after treatment with I5 or I6 was calculated (Fig. 2a-b). Normalized OCR and key parameters of mitochondrial function, such as basal and maximal respiratory capacity, spare respiratory capacity (SRC) and ATP-production coupled to respiration were all affected by I5 and I6. SRC reflects the differences between maximal respiration and basal OCR, indicating that cells treated with I5 and I6 are less able to overcome ATP demands under different types of mitochondrial stress. To further analyze the metabolic phenotype of treated cells, we assessed cell viability in OXPHOS-independent and OXPHOS-dependent conditions: in the presence of Glucose (OXPHOS-independent conditions) or Galactose (OXPHOS-dependent conditions)^37^. A similar reduction in cell viability was observed in glucose and galactose cultures, suggesting that I5 and I6 affect overall cellular metabolism (i.e., both glycolysis and OXPHOS) (Fig. S2a). In support of this claim, global ATP and lactate levels (Fig. S2b, c) were significantly lower following treatment, with I5 showing a more potent effect than I6 on lactate inhibition. Indeed, I6 had no apparent effect on ECAR (Fig. S1b, c), while I5 had a strong effect on both OCR and ECAR (Fig. S1b, c upper panels). Finally, using OCR and ECAR to generate energetic plots, a clear switch in the metabolic profile of cells treated with I5 and I6 to a more quiescent profile, especially for I5, was observed (Fig. S1b, c bottom panels).

### Iodido prototypes induce cellular senescence in GI cancer cells

As mentioned above, mitochondrial dysfunction triggers apoptosis, and senescence^38–40^. While we have already described the apoptotic effect of these iodido agents in the MKN45 cell line^15^, in this study, we further evaluated their toxicity across all GI cell lines, measuring additional parameters. While CDDP induced the phosphorylation of CHK1/2, I5 and I6 complexes did not activate this signaling pathway indicating that these agents do not principally use the DNA Damage Response (DDR) pathway (Fig. S3a). CDDP mainly induces apoptosis via a mitochondria-intrinsic apoptosis pathway, via the pore-forming effector BCL2-family proteins^41^. None of the BCL2-family proteins measured were markedly affected after treatment with the iodido prototypes. Only a transient expression of MCL1 and a decrease in the BID full length protein was observed (Fig. S3b). Together, these data suggest that the effect of these agents on viability does not depend exclusively on the induction of apoptosis.

**Fig. 3:**
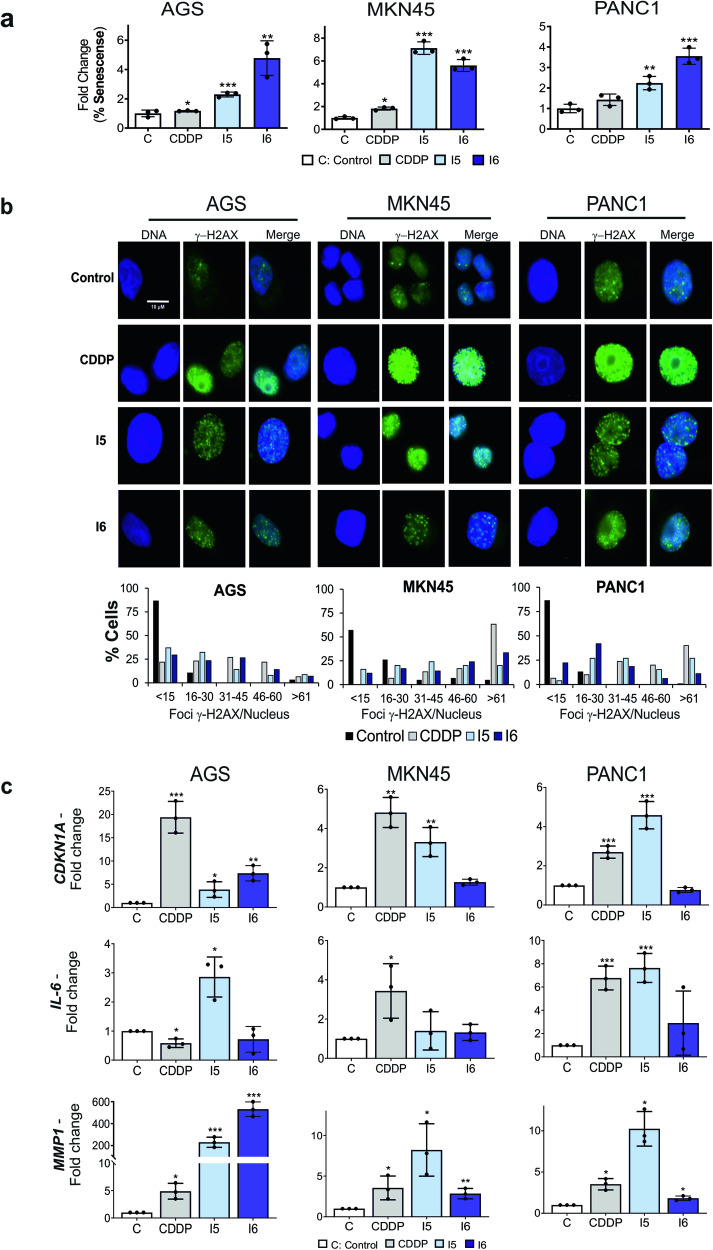
Iodido agents induce cellular senescence in GI cancer cells. **a** Mean fold change ± SD in CellEvent Senescense Green Probe detection, determined by flow cytometry, in AGS, MKN45 and PANC1 cells treated with CDDP, I5 or I6 IC_50_ doses for 24 h. *p < 0.05, **p < 0.01, ***p < 0.001, as determined by unpaired two-sided Student’s *t*-test, compared to untreated (C) set as 1.0. **b** AGS, MKN45 and PANC1 cells were treated with CDDP, I5 or I6 (IC_50_ concentration, see Methods) for 3 h. γ-H2AX foci (green fluorescence) were detected by immunofluorescence using DAPI to stain nuclear DNA (blue fluorescence). Representative images of each condition were taken. Graphs represent the percentage of nuclei with <15, between 16 and 30, between 31 and 45, between 46 and 60 and >60 γ-H2AX foci per nuclei for each condition. Data represent the mean values obtained in three experiments performed in duplicate. **c** RNA was isolated from AGS, MKN45 and PANC1 cell lines stimulated with a 24 h treatment of the complexes. *CDKN1A*, *IL-6* and *MMP1* were quantified by RT-qPCR. Target gene expression was normalized to *GAPDH*. All the experiments were performed three times with IC_50_ concentrations of each compound used in all the assays. The statistical significance was evaluated with Student’s 2-tailed *t*-test (*p < 0.05, **p < 0.01, ***p < 0.001) compared to untreated (C) set as 1.0.

Since CDDP triggers cellular senescence in some cell types, we next evaluated the capacity of the I5 and I6 complexes to induce senescence, as an alternative mechanism to explain cell death. To do that, we assessed the expression of the senescence-associated β-galactosidase (SAβGAL), one of the most common hallmarks of senescence, by flow cytometry or by a SAβGal colorimetric assay^22,42,43^. First, AGS, MKN45 and PANC1 cells were incubated for 24 h with the compounds and the percentage of senescent cells was calculated using a flow cytometry-based assay. Secondly, to evaluate the effect of each compound at early stages, AGS cells were incubated with H_2_O_2_, CDDP, I5 and I6 for 3 h, and then stained with an SAβGal colorimetric kit (see Methods). We observed that both iodido complexes increased the percentage of senescent cells in both assays, even more efficiently than CDDP, with a stronger increase in the SAβGal-positive population produced with I6 (Fig. 3a and Fig. S4a).

We next analyzed the DNA double-strand breaks in AGS, MKN45, and PANC1 cells as they are often associated with senescence^44,45^. Quantification of H2AX^Ser139^ and 53BP1 positive nuclei (Fig. 3b and Fig. S4b) showed that the number of foci per cell significantly increased 3 h after treatment (but not as much as CDDP) on a range of 16–45 foci per nuclei. In the case of 53BP1, we observed that the number of foci increased in the 16–30 range in AGS and PANC1 cells. In both examples, the effect of the iodido prototypes was not as a strong compared to CDDP, whose main target is nuclear DNA.

**Fig. 4:**
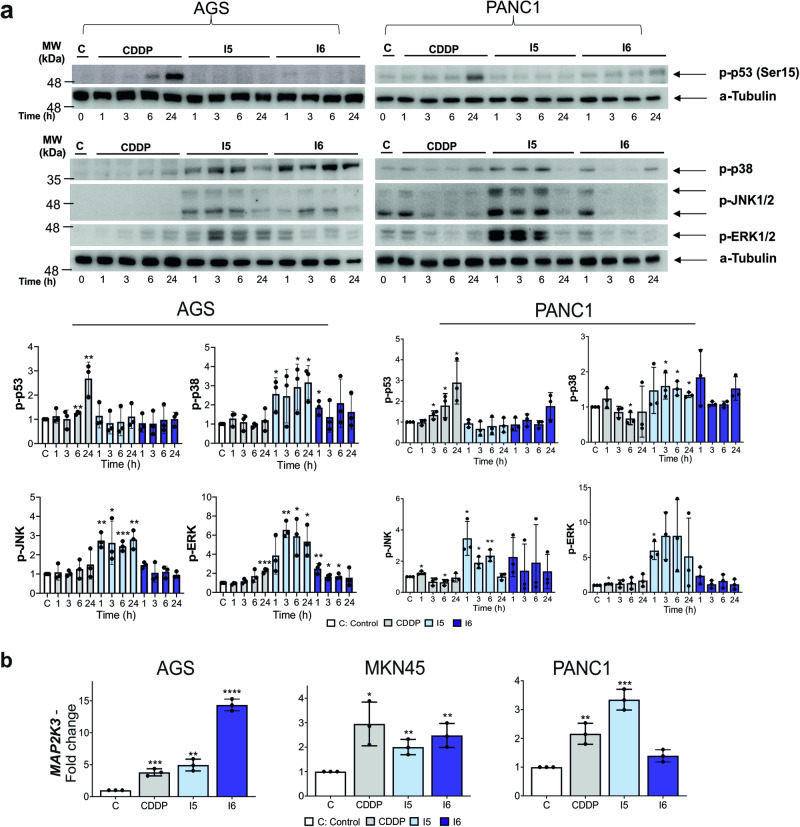
A stress signaling pathway is activated after treatment with iodido prototypes. **a** Top: Representative western blots of phosphorylated forms of p53, JNK, p38 and ERK, in AGS and PANC1 cells after the treatment with IC_50_ concentration of CDDP, I5 or I6 at different times (1, 3, 6 and 24 h). α-Tubulin was used as an endogenous loading control. Bottom: Graphs show the mean ± SD densitometric analyses of each protein normalized with α-tubulin from three independent experiments by using ImageJ (Area under the peak method), Control cells (C, white bars), CDDP (gray), I5 (light blue) and I6 (dark blue). **b** RNA was isolated from AGS, MKN45 and PANC1 cell lines stimulated with a 24 h treatment of the complexes. *MAP2K3* expression levels were quantified by RT-qPCR and normalized with *GAPDH*. Shown are the mean fold change ± SD from triplicate samples (*n* = 3). The statistical significance was evaluated with Student’s 2-tailed *t*-test (*p < 0.05, **p < 0.01, ***p < 0.001, ****p < 0.0001) compared to the untreated cells (C: Control) set as 1.0.

Our next step was to analyze other senescence molecular markers such as p53, p21, Ki67, TNFRSF10D, and the two SASP proteins IL6 and MMP1^46^. I5, I6 and CDDP increased the expression of *CDKN1A* (p21), *IL6* and *MMP1* in the three cell lines, although levels were variable between cell lines (Fig. 3c). We also observed an increase in *TNFRSF10D* and a reduction in *MKI67* levels (Fig. S4c). WB analysis showed that I5 and I6, in contrast to CDDP, did not activate p53 (Fig. 4a), also demonstrated in MKN45 cells^15^. Moreover, activation of MAPKs has also been associated with senescence. We observed that I5 robustly activated p38, JNK and ERK1/2 with transient kinetics, reaching maximum levels at ~6 h. I6 also increased p38 phosphorylation in a persistent manner throughout time, and transiently activated JNK and ERK1/2 (Fig. 4a). RT-qPCR analysis confirmed that all drugs (CDDP, I5 and I6) increased the expression of *MAP2K3*, the kinase that phosphorylates p38, 24 h after treatment (Fig. 4b). The sum of these data suggests that p38 could be involved in I5- and I6-senescence induction.

### The iodido prototypes generate ROS in GI cancer cells

**Fig. 5:**
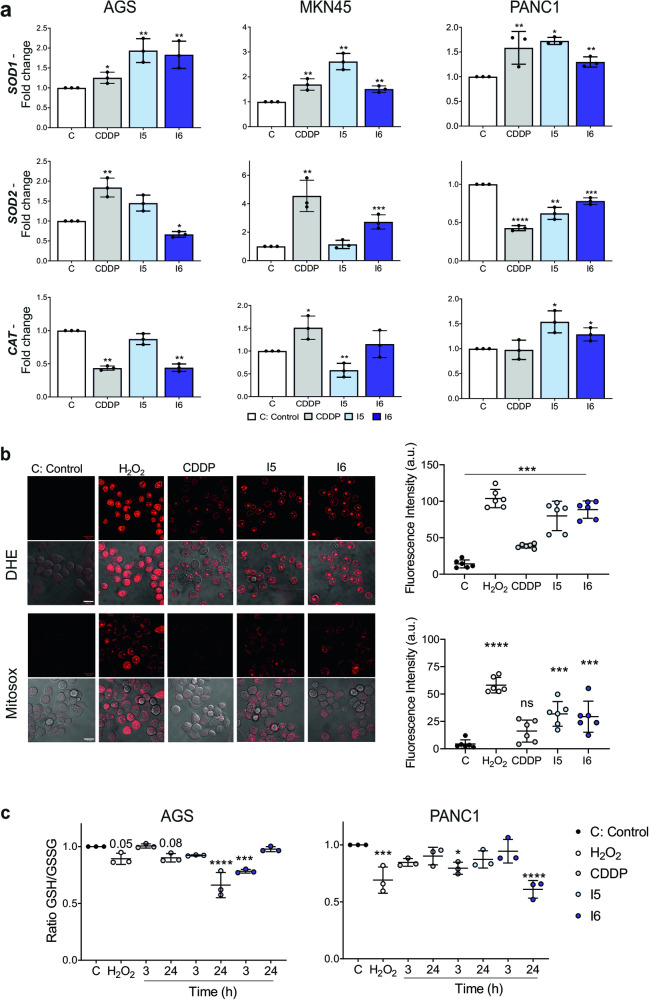
Iodido compounds modulate redox cell response and generate ROS in GI cancer cells. **a** RNA was isolated from AGS, MKN45 and PANC1 cell lines stimulated with a 24 h treatment of CDDP or the iodido complexes (IC_50_ concentration, see Methods). *SOD1, SOD2 and CAT* were quantified by RT-qPCR. Target gene expression levels were normalized with *GAPDH*. Shown are the mean fold change ± SD from triplicate samples (*n* = 3). **b** Detection of ROS in MKN45 cells after the treatment with H_2_O_2_ (200 µM) and the IC_50_ concentration of CDDP, I5 or I6 by confocal microscopy using DHE and MitoSox as O_2_^•−^ fluorescence indicator, in cytosol and mitochondria, respectively. Cells were treated with the compounds for 1 h followed by 30 min of incubation with the probes. Left: Representative images of each condition were taken. Scale bar represents 20 μm. Right: Mean fluorescence intensity ± SD (per cell) was quantified and depicted in the graph. The experiment was performed three independent times in duplicate. a.u. = arbitrary units. **c** Ratio of GSH/GSSG determined with a commercial colorimetric kit (see Methods). AGS and PANC1 cells were treated with the IC_50_ concentration of the compounds and were collected after 3 h and 24 h to perform the assay. *n* = 3. Shown is the ratio of GSH/GSSG ± SD. In all the experiments, statistical significance was evaluated with Student’s 2-tailed *t*-test (*p < 0.05, **p < 0.01, ***p < 0.001, ****p < 0.0001) compared to the untreated cells (C: Control) set as 1.0.

Mitochondrial dysfunction can also increase cellular ROS. Alterations in the electron transport chain, whose final substrate is O_2_, are the main source of intracellular ROS. Moreover, oxidative stress and increased ROS levels could act as inducers of senescence. Since CDDP has been reported to induce oxidative stress in various cell types^47^, we investigated whether the iodido prototypes generate ROS in vitro. As a first approach, we measured variations in different detoxification enzyme-coding genes following treatments with CDDP, I5 and I6, to determine if these agents could be acting as ROS generators. Indeed, a strong induction of *SOD1* was observed after 24 h with all the treatments (Fig. 5a) suggesting O_2_^·-^ generation across all the cell lines tested. The expression of *SOD2* and *CAT*, however, was not significantly affected after treatment. The results encouraged us to continue studying ROS generation and associated effects with the iodido prototypes. Accordingly, the generation of cellular ROS in response to treatment with CDDP, I5 and I6 was examined. MKN45 and PANC1 cells were incubated with CDDP, I5 and I6 for 1 h and the levels of cytosolic and mitochondrial superoxide anions (O_2_^·−^) were measured in live cells (See Methods for details). Incubation with 200 µM H_2_O_2_ for 1 h (positive control) rapidly increased fluorescence of DHE and Mitosox, compared with untreated cells. Similarly, the compounds significantly increased fluorescence with respect to untreated cells, being higher for prototypes I5 and I6 than for CDDP (Fig. 5b and S5). Finally, we investigated the GSH/GSSG system to elucidate the cellular redox status after treatment with the complexes. The ratio of reduced GSH to oxidized GSH (GSSG) is an indicator of cellular health. A lower GSH/GSSG ratio indicates a higher amount of ROS in cells. We observed that PANC1 and AGS cells treated with CDDP, I5 and I6 decreased the GSH/GSSG ratio, over time, similar to that obtained after H_2_O_2_ treatment (positive control) (Fig. 5c). These results strengthen the evidence that the iodido prototypes can effectively generate ROS and probably oxidative damage associated with these harmful radicals, even in a more efficient way than CDDP.

### NAC has a protective effect in GI cell viability after treatment with iodido complexes

N-acetylcysteine (NAC) has been widely reported to have a strong protective effect against oxidation damage in cultured cells by acting as a general ROS scavenger. Thus, we tested the ability of CDDP, I5 and I6 complexes to affect cellular viability in the presence or absence of a non-toxic concentration of NAC (0.5 mM), which has been used in different publications for a similar purpose^48,49^. As expected, NAC treatment reversed the toxic effects of the iodido drugs, but not CDDP (Table 1, Fig. S6a). We confirmed this effect at the level of cell proliferation by using a colony formation assay. Specifically, an increase in the number of CFUs (colony forming units) was obtained in those plates that combined NAC with I5 or I6 but not with CDDP (Fig. S6b), indicating that anti-tumor effects of the iodido complexes can be due mostly to ROS induction and oxidation damage, which can be rescued by scavenging ROS with ROS scavengers like NAC.

**Table 1:** IC_50_ concentration ( ± SD) at 48 h in the presence and absence of NAC (0.5 mM)

	AGS		MKN45		PANC1	
	NAC (-)	NAC (+)	NAC (-)	NAC (+)	NAC (-)	NAC (+)
**CDDP**	26.67 ± 2.21	22.65 ± 4.03	9.45 ± 1.10	10.23 ± 1.56	12.33 ± 1.16	9.46 ± 1.29
**I5**	38.19 ± 3.50	>100	28.70 ± 1.03	>100	18.16 ± 5.06	>100
**I6**	51.52 ± 3.68	>100	56.75 ± 1.53	>100	18.84 ± 10.29	>100

N = 3

### I5 reduces in vitro clonogenicity and inhibits in vivo tumor growth with no systemic toxicity

Systemic toxicity to normal cells is one of the main obstacles in platinum chemotherapy with severe side effects due to the lack of specificity. The fibroblast cell line CC2509 and the Human Pancreatic Duct Epithelial cells (HPDE) were used as in vitro cell culture models to evaluate the potential cytotoxicity of the complexes in non-cancer control cells compared with tumor cells and with CDDP. Both complexes displayed moderate toxicity, higher in HPDE than in the fibroblasts, but markedly lower than CDDP (Fig. S7a, Table 2). Specially I5 showed low toxicity in CC2509 (>70 µM) and the IC_50_ in HPDE was higher than in tumor PANC1 cells (26 vs 18 µM). Cell proliferation was evaluated with a CFU-assay to assess the capability of PANC1 to form clones. To this end, we exposed PANC1 cells for 1, 3 and 24 h to CDDP, I5 and I6 and after 10 days colonies were quantified. We observed a marked decrease in the clonogenic capacity of PANC1 cells following treatment, even with as little as 1 or 3 h of treatment. As the effect with I5 was more potent compared to I6 (Fig. S7b), we next performed in vivo proof-of-concept studies to validate the anti-tumor properties of I5 in xenograft mouse models.

**Table 2:** IC_50_ concentration ( ± SD) at 48 h in non-tumoral cell lines

	HPDE	CC2509
**CDDP**	0.45 ± 0.05	23.82 ± 9.55
**I5**	26.83 ± 2.43	74.13 ± 5.42
**I6**	14.25 ± 5.37	33.81 ± 6.24

N = 3

**Fig. 6:**
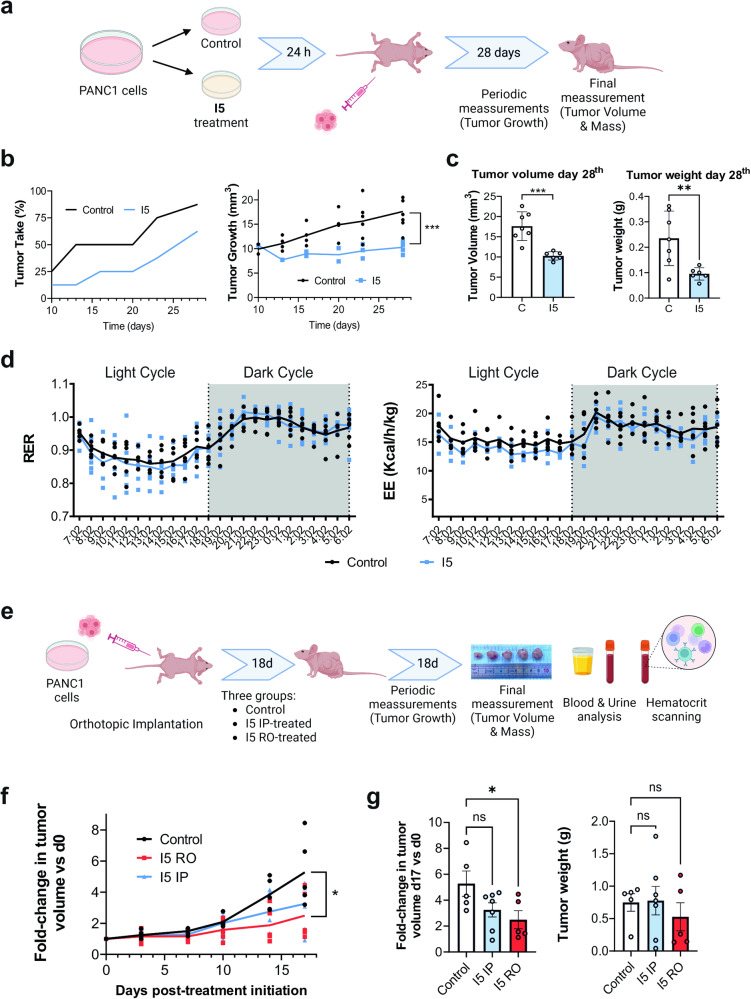
I5 reduces in vivo PANC1 tumor growth. **a** Summary of the in vivo experimental design for the pre-treated PANC1 cell xenograft in vivo studies. Schematic created with BioRender.com. **b** Growth curves indicating tumor take (left, defined as no. tumors formed/no. of injections at the indicated time point) or the mean tumor volume (mm^3^) (right) ± SD over 28 days following injection of 1 × 10^5^ Control diluent-treated or I5-treated PANC1 cells. (no. of injections = 8–9). **c** Quantification of the mean tumor volume and mean weights (**g**) ± SD for control (**c**) and I5-pre-treated tumors (no. of injections = 8–9). Statistical significance was evaluated with Student’s 2-tailed *t*-test (**p < 0.01, ***p < 0.001). **d** Indirect calorimetry analyses of mice treated with I5. Left: Respiratory exchange ratio (RER) was determined as V_CO2_/V_O2_ and right: Energy expenditure (EE) was calculated as (3.185 + 1.232 x RER) x V_O2_. Shown are the mean RER and mean EE (Kcal/h/Kg) values ± SD for mice implanted treated intravenously with I5 (1.4 mg/Kg) or physiological saline (i.e., Sham) as a function of time (24 h). **e** Summary of the in vivo experimental design for the treatment of mice harboring PANC1 xenografted tumors. Schematic created with BioRender.com. **f** Average fold change in tumor volume ± SEM in mice bearing PANC1 xenografts and treated with diluent control (Control), I5 (1.4 mg/kg i.p. or r.o.; 3-times per week) and compared to d0 (*n* = 5-7 tumors/group). **g** Mean fold change in tumor volume ± SEM (left) or tumor weight ± SEM (right) determined at treatment cessation. *p < 0.05, as determined by one-way ANOVA with Dunnett post-test, compared to Control. ns not significant; g gram; d0 day 0.

As a first approximation, PANC1 cells were treated for 24 h with I5 (20 µM) and then injected into immunocompromised nude mice (Fig. 6a). In parallel, we also injected PANC1 diluent-treated control cells. Every 2–3 days for 4 weeks, we monitored tumor take and tumor growth in both treatment arms, and on day 28 mice were sacrificed and the tumor volume and weight of the tumors formed were determined. Not only did we observe a clear delay in tumor take and growth in the treatment arm throughout the course of the experiment (Fig. 6b), but the tumors that did form following pre-treatment of cells with I5 had significantly reduced volumes and weights (Fig. 6c). These promising results encouraged us to perform direct in vivo treatments with I5; however, we first tested the safety of I5 in vivo. To this end, C57Bl6 mice were treated with a maximum feasible dose of I5 (1.4 mg/kg), administered via tail vein injection, and mice were weighed and monitored. Importantly, no adverse effects on weight nor on subtle neurological perturbations (as determined by Irwin’s test) were observed at early acute time points. Next, an indirect calorimetry analysis was performed, and no differences were observed between treated and untreated mice at the level of Respiratory Exchange Rate (RER), Energy Expenditure (EE), Locomotor activity, nor on food or water intake (Fig. 6d and S8), illustrating that, under these conditions, I5 is non-toxic in vivo. With these safety results in hand, mice subcutaneously implanted with PANC1 tumors (see Methods for details) were randomized and administered I5 i.p (1.4 mg/kg, daily) or r.o. (1.4 mg/kg, daily) (Fig. 6e). Monitorization of tumor volume by caliper measurement revealed that I5 i.p. and r.o. administration reduced tumor growth over the course of the experiment, with r.o. administration showing a statistically significant effect on tumor volume and a marked reduction in tumor weight at the conclusion of the experiment (Fig. 6f-g). Importantly, following 17 days of daily I5 treatment, no adverse effects on hematocrit parameters nor on plasma blood and urine analyses were observed (Fig. S9 and Table S1–S2), indicating again that I5 has anti-tumor activity with no associated in vivo toxicity.

## Discussion

Since the discovery of CDDP, interest in developing new platinum agents has increased, focusing on minimizing the severe side effects and the inherent resistance associated with CDDP. Among those developed, different iodido complexes have shown the capacity to overcome CDDP resistance (intrinsic or acquired) in different cancer cell lines^14^, including our studies with I5 and I6^11–13^. Herein we build upon these studies and show that I5 and I6 also modulate tumor cell metabolism by reducing OXPHOS and glycolysis and increasing ROS and cellular senescence. Our detailed analyses of the mechanism of action of these iodido agents indicate a triple accumulative loop with three interconnected processes: (i) mitochondrial dysfunction and metabolic disruptions; (ii) ROS generation and oxidative damage; and (iii) cellular senescence. To the best of our knowledge, these are the first mechanistic studies related to the biomolecular action of platinum iodido compounds, revealing their safety in vivo and their potent anti-cancer activity in xenograft assays. Thus, I5 and I6 could be considered potential chemotherapeutics for GI tumors and should be further developed and studied.

We previously described that I5 and I6 induce apoptosis in GC MKN45 cells partially independent of p53, which is a mechanism of action opposite of CDDP^15^. In this work, we further confirm a p53-independent mechanism of action for I5 and I6 in two additional GI cell lines (AGS, and PANC1). Neither I5 nor I6 induced *PUMA* or *BAX*, which are p53 target genes^50,51^, results that are in agreement with other publications with iodido complexes^14^.

The most extended target of CDDP and derivatives is nuclear DNA. The ability of CDDP to affect the mitochondria and, in particular, mtDNA has also been reported^52^. In fact, recent reports highlight the importance of ROS generation and oxidation damage associated with these platinum-based treatments in cell death^4,53^. Comparable with CDDP, I5 and I6 caused significant oxidative damage of genomic and mtDNA. As such, we studied the mitochondrial status in treated cells, detecting a decrease in the ratio ΔΨ_m_/Mit. Mass suggesting mitochondrial perturbations. Mitochondrial damage was higher than CDDP (regarding ΔΨ_m_) pointing to the mitochondria as a potential target for I5 and I6.

CDDP-resistant cancer cells can evade drug toxicity by reprogramming their metabolism^54^. As such, metabolic plasticity of cancer cells has become a new challenge for cancer therapy^17,20,55–57^. We demonstrated that both I5 and I6 disrupted the metabolism of treated cells; however, when comparing both compounds, we observed differences in their mode of action regarding cell metabolism. I6 modulates OXPHOS while glycolysis seems to be less affected. I6-based therapies could be advantageous in those tumor cells where OXPHOS is the main route of obtaining ATP, such as in cancer stem cells^37,58,59^. On the other hand, the trans prototype I5 achieved a promising hypometabolic state. This scenario is very advantageous as tumor cells can undergo metabolic plasticity, compensating OXPHOS with glycolysis and vice versa in order to meet their energetic ATP requirements^60,61^. Moreover, the presence of hypoxic regions is also common in a majority of solid tumors, where proliferative cancer cells only obtain ATP *via* glycolysis. Thus, by inhibiting both metabolic pathways (OXPHOS and glycolysis), I5 stands out as a very attractive metabolic modulator, as it could target both glycolytic and OXPHOS-dependent cancer cells. Indeed, a main focus in chemotherapy rational designs lies in searching for new compounds able to target multiple metabolic weaknesses and/or the metabolic plasticity/heterogeneity of tumor cells^62^. I5, and to a lesser extent I6, meet these requirements.

Physiologically, we also show that I5 and I6 induce senescence more efficiently than CDDP, which has been widely reported as a senescent inductor in different carcinomas: Lung^63^, Ovarian^64^, Melanoma^65^, and also GC^44^. In contrast to CDDP and other platinum-based drugs, the senescence induced by I5 and I6 was shown to be independent of p53; however, we did observe an increase in the expression of *CDKN1A* (gene that encodes p21 protein, commonly associated with p53 activation) that could be induced by other transcription factors (ELK1, SP1) via MAPKs^66,67^. Related to the SASP, we observed a strong increase in MMP1 expression, and also IL-6 showed a clear induction after I5 and I6 treatments. There is still a controversy between the benefits of using pro-senescent drugs in cancer treatment, generally due to the dual role of SASP^23,68^. On the one hand, SASP could favor tumor progression, while on the other hand, SASP can activate the inflammatory response and the immune system to eliminate tumor cells. There are recent reports supporting the advantages of using a combinatorial therapy-based approach, with a first line treatment consisting in a chemotherapy that induces senescence in cancer cells, followed by a second line treatment with a senolytic that selectively kills these senescent cells^24,69^. Although further studies will have to be carried out, SASP seem to have a key role in the cellular senescence provoked by I5 and I6. Likewise, further studies using I5 or I6 in combination (or sequentially) with standard chemotherapy are still needed.

Mitochondrial dysfunction is considered a general feature of cellular senescence, usually related with an increase in ROS levels and oxidative damage^39^. The electron transport chain is the last step in OXPHOS, with molecular oxygen being the final electron-acceptor. Thus, modulation of mitochondrial metabolism can lead to an enhancement in ROS levels, which can disrupt different signaling networks and generate a wide array of harmful radicals leading, in many cases, to cell death^70^. The presence of ROS activates the cell stress response, usually via MAP kinases. Many MAP kinases, especially ERK1/2, can also be activated by the SASP^65^. Accordingly, I5 and I6 induced the phosphorylation of ERK1/2, and also p38 and JNK1/2, suggesting a common scenario in senescence associated with oxidative damage^71,72^. In addition, it is widely reported that senescent cells have increased ROS levels^27,73^.

Biological organisms have developed different strategies to confront redox injuries caused by endogenous or exogenous stimuli. We did not observe a proper detoxification mechanism, only detecting *SOD1* overexpression after treatment with I5 or I6. This imbalance could increase oxidation damage in different organelles, which could lead to cell death. We used NAC, a well-stablished antioxidant and ROS scavenger, to support this claim^74,75^. While CDDP activity remained unaltered, we detected a clear protective effect in cells when NAC was combined with I5 or I6. One concern associated with metallodrugs studies and model proteins is the direct interaction between them. Our group, however, has previously demonstrated that the interaction between these iodido compounds (especially I5) and NAC and other sulfur donor model proteins were lower than in the case of CDDP^12,13^. So, we can conclude that the upregulation in ROS caused by I5 and I6 could be responsible of their physiological effect.

Cultured cells are useful, economic, and powerful tools for initial wide screenings of the pharmacological activity and properties of new drugs; however, candidate compounds still need to be studied in other more complex experimental models, such as 3D cultures (e.g., organoids) or animal models, to truly appreciate their potential and future clinical applicability. Indeed, additional in vivo or 3D-based experiments are needed, but our pilot in vivo studies with xenografted cells shows proof-of-principle that I5 has strong anti-cancer properties in vivo. Specifically, I5 showed a potent effect on the growth of pre-treated PANC1 cell in vivo and in vivo in I5-treated mice with already established PANC1 xenografts, resulting in decreased overall tumor mass and volume. Furthermore, I5 had no systemic toxicity, within the parameters tested, which included energy balance, behavior, blood and urine analyses. To the best of our knowledge, this is the first report of this type of iodido agent being safely administered in vivo to treat a GI cancer. These promising anti-tumor results therefore position I5 as an emerging and very promising antitumorigenic metallodrug, which should be further studied in other preclinical tumor models.

Finally, we summarize our work by establishing a triangular interaction between the aforementioned processes. Oxidative damage and the increase in ROS levels could be the cause of senescence and mitochondrial perturbations. However, it is also feasible that cellular senescence produces an increase in dysfunctional mitochondria, and altogether results in higher ROS generation. Another possibility is that mitochondrial and metabolic disruption could trigger senescence and enhance the concentration of cellular ROS. We cannot assess which process occurs first, nor which has a prominent role in toxicity, but altogether, the effects are quite potent in GI cancer cells, and in PDAC xenografts in vivo. Despite the advances in new therapies, chemotherapy continues to be the most used alternative. Consequently, the development of new antitumor metallodrugs able to overcome different clinical barriers in platinum-based chemotherapy is a public healthcare priority. In comparison with CDDP, the clinical interest in these prototypes lies in: (i) their greater solution stability^11^; (ii) their lower interaction with sulfur and serum proteins, minimizing deactivation processes^12^, and (iii) their greatest advantage being their ability to overcome CDDP resistance^13,14^. Despite the structural analogy between the complexes, the isomerism has a key role in their final activity and mechanism of action. Both iodido agents generate ROS and oxidative damage, which finally affects cell viability. Although both compounds induce cellular senescence, I6 seems to have a more profound effect emerging as a pro-senescent compound, that in combination with a second senolytic treatment could potentiate its effects. On the other hand, I5 has demonstrated an important effect on cellular metabolism, achieving a hypometabolic state, which is of great interest due to the metabolic plasticity of cancer cells. In theory, I5 would affect both metabolic pathways (i.e., glycolysis and OXPHOS), almost inhibiting total ATP production, leading to cell death in those cells that depend on glycolysis and in those cells that are OXPHOS-dependent, like CSCs. This fact could be responsible for its high activity in decreasing colony formation and tumor growth in vivo. Importantly, while I5 appears to be a global metabolic inhibitor, in vivo we observed no adverse or toxic effects, boding well to its possible clinical utility. In summary, the promising results described herein for I5 (and I6) are encouraging and warrant further studies in additional in vivo models to translate these results to the clinical setting.

## Methods

### Cell culture

The human gastric adenocarcinoma cell lines MKN45 (poorly differentiated; DSMZ: Deutsche Sammlung von Mikroorganismen und Zellkulturen GmbH), and AGS (primary tumor; ATCC/LGC Standards, Spain) were cultured in RPMI 1640 (Sigma) and HAM’s F-12 + AA’s (Gibco), respectively, according to the specifications of the manufacturer’s datasheet. The pancreatic cell line PANC1 (ATCC) was cultured in RPMI 1640 (Sigma). Control normal cell lines CC2509 (fibroblasts, ATCC) and HPDE (Human Pancreatic Duct Epithelial cells, ATCC) were cultured in RPMI and DMEM/F12 (Gibco), respectively. HAM’s F-12 and RPMI were supplemented with 10% Fetal Bovine Serum, 2 mM L-Glutamine, Fungizone (2.5 μg/mL) and Gentamicin (0.035 mg/mL). DMEM/F-12 was supplemented with B-27 (10889038, Gibco), 2 mM L-Glutamine, Fungizone (2.5 μg/mL), Gentamicin (0.035 mg/mL) and 20 ng/mL bFGF (PeproTech). Cells were cultured at 37 °C, 5% CO_2_, and 95% humidity. Mycoplasma contamination tests are frequently run in our laboratory. Experiments were performed between passage two to eight.

### Chemicals

CDDP was supplied by Ferrer FARMA. The compounds used for the assays (I5 and I6) were synthesized following previously reported procedures^15^. In all the experiments cells were treated with the metallodrugs following the appropriate sample preparation: CDDP was dissolved in water, I5 and I6 in DMSO and then immediately diluted with culture medium to the appropriate concentration, with a final DMSO concentration of 1%. Control conditions also contained 1% of DMSO.

### Cell viability

Cell viability was assessed using a crystal violet-based staining method. IC_50_ concentrations were calculated at 48 h. (i) AGS: CDDP 25 µM, I5 40 µM, I6 50 µM; (ii) MKN45: CDDP 10 µM, I5 30 µM, I6 50 µM; (iii) PANC1: CDDP 10 µM, I5 20 µM, I6 20 µM (Fig. S6a); (iv) HPDE: CDDP 0.5 µM, I5 25 µM, I6 15 µM; (v) CC2509: CDDP 25 µM, I5 75 µM, I6 35 µM (Fig. S7 and Table S1). The cells (2 × 10^5^) were seeded in 24-multiwell dishes in 0.5 mL of complete medium and incubated overnight. To evaluate the role of ROS in cytotoxicity, the growth medium was supplemented with NAC 0.5 mM (a reported non-toxic concentration^48,49^), a general ROS scavenger. After 48 h, cells were fixed with 1% glutaraldehyde (20–30 min), washed with distilled water, and stained with 0.1% crystal violet. A colorimetric-based assay set at 595 nm was used to estimate the number of cells per well for CDDP, I5 and I6. IC_50_ were calculated by using GraphPad Prism (version 8.0). Nonlinear regression was used to fit the data to the log (inhibitor) versus response (variable slope).

### DNA extraction and quantification of oxidized bases in specific genome regions by qPCR

Total cellular DNA from MKN45 and PANC1 cells was extracted using an NZY Tissue gDNA Isolation Kit (Nzytech, MB13503) following the manufacturer’s instructions and quantified using a Nanodrop (Nanodrop Spectrophotometer ND-1000, ThermoFisher Scientific, Wilmington, DE, USA). We adapted the procedure described by O’Callaghan et al.^76^ to measure oxidative DNA damage at telomeres, 36B4 genomic region and at mitochondrial DNA (MT-COX1, MT-CYB). This is a qPCR method based on differences in PCR kinetics between DNA template digested by Formamidopyrimidine DNA Glycosylase (FPG, Cat no. M0240S) and undigested DNA. This enzyme recognizes and cuts 8-oxoguanine, producing apurinic sites that are converted into single-strand breaks by its apurinic sites lyase activity. These single-strand breaks inhibit the PCR, thus, the ΔCT after digesting DNA by 8-oxoguanine (Ct digested–Ct undigested) is proportional to the oxidative damage in the amplified region. DNA was incubated with 8 Units of FPG during 3 h in FPG buffer (NEB1, M0240S). The reaction was stopped by incubation at 95 °C for 5 min. qPCR analysis was performed on 40 ng of digested or undigested genomic DNA. Each qPCR was performed in triplicate including no-template controls in a StepOne Real‐time PCR System (Applied Biosystems, 4376357). Target genes (Table 3) were amplified using SYBR Green (Applied Biosystems, MB345). Thermal cycling of the qPCR reaction was initiated with a denaturation step at 95 °C for 10 min. The process consisted of 40 cycles (denaturation at 95 °C for 15 sec, annealing at 60 °C for 30 sec, and elongation at 72 °C for 30 sec). This experiment was repeated three independent times with 10–12 replicates.

**Table 3:** Primer sequences for the quantification of oxidation DNA damage assay

Primer	Sequence
36B4	F: CAGCAAGTGGGAAGGTGTAATCC R: CCCATTCTATCATCAACGGGTACAA
Telomere	F: CGGTTTGTTTGGGTTTGGGTTTGGGTTTGGGTTTGGGTT R: GGCTTGCCTTACCCTTACCCTTACCCTTACCCTTACCCT
MT-COX1	F: CACAGCCCATGCATTTGTAA R: GATGCGAGCAGGAGTAGGAG
MT-CYB	F: CCACCCTCACACGATTCTTT R: TGGCTTAGTGGGCGAAAT

### Flow cytometry

We used flow cytometry to study the mitochondrial mass, mitochondrial membrane potential (ΔΨ_m_) and cellular senescence of untreated and treated cells. Cells (5 × 10^4^) were seeded in 24-multiwell dishes and cultured overnight. Then, cells were treated for 24 h with the IC_50_ concentration of each compound. Cells were tripsinized, stained with Mitoprobes and resuspended in Flow buffer [1X PBS; 3% Fetal Bovine Serum (v/v); 3 mM EDTA (v/v)] before analysis with a 4-laser Attune NxT Acoustic Cytometer (Thermo Fisher Scientific). For Mitoprobe assays, 2 mg/ml DAPI (Sigma) was used to exclude dead cells. Data were analyzed with FlowJo 9.3 software (Tree Star Inc., Ashland, OR). For mitochondrial mass and ΔΨ_m_ measurement, Mitotracker Green (MTGreen, M7514, Invitrogen) and Mitotracker Red CMXRos (CMXROS, M7512, Invitrogen) were used, respectively. Probes were incubated with cells for 30 min at 37 °C at a concentration of 100 nM (MTGreen) and 20 nM (CMXROS), and fluorescence was detected using the filters (Ex488nm/Em530/30) BL1 and (Ex561nm/Em585/16) YL1, respectively. To determine mitochondria functionality, the ratio between ΔΨ_m_ (MitoTracker Red CMXRos) *versus* Mit. Mass (MitoTracker Green) was calculated. The gating strategy used in all the Flow Cytometry experiments is reflected in Fig.S10.

### Cell metabolism

Oxygen Consumption Rate (OCR), Extra Cellular Acidification Rate (ECAR) and Energy map measurements. Cells were plated in XF HS Miniplates (Seahorse Bioscience) at a cellular density of 1 × 10^4^ cells/well and incubated overnight. Then, cells were treated for 24 h with the IC_50_ concentration of each compound. For OCR determination, cells were incubated in Seahorse XF DMEM media (103680, Agilent) supplemented with 2 mM glutamine, 10 mM glucose, and 1 mM pyruvate for 1 h, prior to the addition of inhibitors (Seahorse XFp Cell Mito Stress Kit, Cat no. 103010, Agilent). After an OCR baseline measurement, the minimum oxygen consumption was determined by adding 1.5 µM oligomycin (O) and the maximal respiration rate was assessed by adding 1 µM carbonyl cyanide p-trifluoro methoxyphenylhydrazone (FCCP, F). At the end of the experiment, the non-mitochondrial oxygen consumption was evaluated by adding both 0.5 µM rotenone (R) and antimycin (A). Experiments were run in a XF HS Mini analyzer (Seahorse Agilent), and raw data were normalized to total protein using a BCA protein assay kit (Cat. no. 23225, Thermo Scientific).

ATP determination assay. Lysate pellets from cells treated with the IC_50_ concentrations of I5 or I6 were collected to evaluate the changes in the levels of ATP. The analysis was performed using the ATP Bioluminescense Assay Kit CLS II (Cat. no. 11699695001, Roche) according to the manufacturer’s instructions. Bioluminescence was determined using a Synergy™ HT Multi-Mode Microplate Reader (BioTek, Winooski, Vermont, USA). Data were normalized to total protein using the BCA protein assay kit (Thermo Scientific).

Lactate production assay. Supernatants from cells treated with the IC_50_ concentrations of I5 or I6 were collected to evaluate the changes in the levels of lactate production. Lactate was measured in the medium harvested from the cells treated 24 h with I5 and I6 IC_50_. Proteins were removed from 50 μL of the medium by adding 100 μL of 8% Percloric Acid and 40% EtOH at 4 °C, and centrifuging at 2 × 10^4^ g for 10 min at 4 °C. The supernatants were then frozen until the lactate in 15 μL of each sample, or lactate standard, was measured mixed with 150 μl of assay buffer (0.5 M glycine [pH 9.5], 0.2 M Hydrazine, 3.4 mM EDTA), 100 μl of H_2_0, 5 μL of 1100 U/mL LDH (Roche) and 30 μL of 15 mM NAD + . NADH production was evaluated by measuring absorbance at 340 nm in a multiplate reader (Synergy HT, Biotek) and it was proportional to the lactic acid concentration in the sample after a 2 h incubation^77^. The lactate concentration was normalized to the total amount of protein measured with the Micro BCA Protein Assay Kit (Thermo Scientific). Assays were performed in triplicate in three independent experiments.

Cell viability after I5 and I6 treatment in glucose or galactose conditions. AGS, MKN45 and PANC1 cells were trypsinized and seeded at a concentration of 5 × 10^4^ cells per well in 24-well plates and cultured in RPMI 1640 containing 10% FBS at 37 °C, 5% CO_2_. After 24 h, cells were treated with the IC_50_ doses of I5 and I6 for 24 h. Subsequently, cells were cultured with either glucose-free Dulbecco’s Modified Eagle Medium (DMEM; Thermo Fisher Scientific) supplemented with 5 mM glucose (0.9 g/l), 10% FBS, 50 units/ml of penicillin and streptomycin, and 1 mM of pyruvate [Glucose: OXPHOS-independent conditions] or glucose-free DMEM medium (Thermo Fisher Scientific) supplemented with 5 mM galactose (0.9 g/L), 10% FBS, 50 units/ ml of penicillin and streptomycin, and 1 mM of pyruvate [Galactose: OXPHOS-dependent conditions]. Sugar concentrations of 5 mM were chosen to mimic physiological sugar levels (glucose, 5 mM) and to avoid potential biological artifacts mediated by supraphysiological sugar levels^37^. After 24 h, cells were fixed with PFA 4% (Paraformaldehyde, 16% w/v aqueous solution, methanol free, Alfa Aesar™, Cat no. 11400580) for 10 min, washed with PBS and stained with Crystal violet (Sigma, Cat no. C3886-100G) for 1 h. Images of wells were digitalized, and cell viability was quantified by lysing stained cells in 1XPBS with 1%SDS followed by colorimetric absorbance analysis using a Synergy™ HT Multi-Mode Microplate Reader (BioTek, Winooski, Vermont, USA).

### Immunofluorescence assay

Cells (7 × 10^4^) were seeded on 20 mm coverslips. After 24 h, cells were challenged (for 3 h) with the IC_50_ concentration of the compounds: CDDP, I5 and I6. Cells were then fixed in 4% formaldehyde for 15 min, washed with PBS, permeabilized with 0.2% Triton for 5 min and finally blocked with 1% BSA for 1 h. Coverslips were incubated for 1 h with the primary antibody (Cell Signaling: H2AX Ser139 (#2595) and 53BP1 (#4937)) at room temperature, followed by a 1 h incubation with the appropriate secondary antibody. DNA was stained with DAPI. Fluorescence microscopy was performed using a NIKON Eclipse 90i. The image analysis was performed using the software program Nikon NIS-Elements and Image J.

### SA-β-Gal activity assays

SA-β-Gal activity was measured following two different approaches. (i) AGS, MKN45 and PANC1 cells (5 × 10^4^) were seeded in 24-multiwell dishes and incubated overnight. Then, cells were treated for 24 h with the IC_50_ concentration of each compound. For flow cytometry cell senescence detection, cells were tripsinized, fixed for 15 min with PFA 4% and stained with the CellEvent Senescense Green Flow Cytometry Assay Kit (C10840, Invitrogen) according to manufacturer’s instructions. CellEvent Senescense Green Probe was used at a 1:500 dilution and fluorescence was detected using the filter (Ex488nm/Em530/30) BL1 with a 4-laser Attune NxT Acoustic Cytometer (Thermo Fisher Scientific). (ii) For histochemical staining assays, AGS cells (2 × 10^5^) were seeded in a 6-well plate, incubated overnight, and treated with CDDP (20 µM), H_2_O_2_ (200 µM) and a IC_25_ concentration of I5 (15 µM) or I6 (15 µM). After 3 h, the treatments were removed, new supplemented medium was added, and cells were incubated for an additional 24 h. Finally, SA-β-Gal staining was performed using the Senescence Cells Histochemical Staining Kit (CS0030-1KT) according to the manufacturer’s instructions.

### RT-qPCR

Total cellular RNA was extracted using Tri-Reagent (Life Technologies), following the manufacturer’s instructions. One μg of total RNA was primed with random primers and cDNA synthesized with an M-MLV reverse transcriptase following the manufacturer’s instructions (Promega). Target genes were amplified using a SYBR Green polymerase chain reaction assay, working with the specific primer sets listed in Table 4.

**Table 4:** Primer Sequences for the RT-qPCR analysis

Primer	Sequence
*MMP1*	F: CTTGCACTGAGAAAGAAGACAAAGG R: ACACCCCAGAACAGCAGCA
*CDKN1A*	F: GCTGCAGGGACAGCAGAG R: GCTTCCTCTTGGAGAAGATCAG
*MAP2K3*	F: CCCCAGTCCAAAGAGAGGCTG R: TTCACTGCGCAGATGGGGACC
*SOD1*	F: GGGCCAAAGGATGAAGAGA R: CTTTCTTCATTTCCACCTTTG
*SOD2*	F: GCTGCTTGTCCAAATCAGG R: CTTTTAGATAATCAGGCCT
*CAT*	F: GCCAGAGCTGTGCAGATGAG R: CAGTGGACAGGTTTCTGACC
*IL6*	F: GCCAGAGCTGTGCAGATGAG R: CAGTGGACAGGTTTCTGACC
*GAPDH*	F: GAGAGACCCTCACTGCTG R: GATGGTACATGACAAGGTGG

Thermal cycling of the qPCR reaction was initiated with a denaturation step at 95 °C for 10 min. The process consisted of 40 cycles (denaturation at 95 °C for 15 sec, annealing at 60 °C for 30 sec, and elongation at 75 °C for 30 sec). PCR amplifications were carried out in a StepOne Real‐time PCR System (Applied Biosystems, 4376357). Relative mRNA levels were calculated by the delta-Ct method (2-ΔΔCt), where each 1-Ct difference equals a two-fold change in transcript abundance, using GAPDH as an endogenous gene reference. ΔΔCT represents the difference between the mean ΔCT value of the cells tested and the mean ΔCT value of the calibrator, both calculated for the same PCR run.

### Western Blotting

Total protein extracts were obtained by adding the lysis buffer: 25 mM HEPES pH 7.5, 0.3 M NaCl, 1.5 mM MgCl_2_, 0.2 mM EDTA, 0.5 mM DTT, 20 mM β-glicerophosphate, 0.1 mM Na_3_VO_4_, 0.1% triton X-100; in the presence of protease inhibitors^78^. Twenty μg of protein per sample were loaded and resolved using 8%, 10% or 15% SDS-PAGE polyacrylamide gels, and then transferred onto PVDF membranes, followed by immunodetection using appropriate antibodies, purchased from Santa Cruz Technology: Mcl-1 (sc-819), Cell Signaling Technology: BIM, BID, BAX (#9942), p-p38 (1:2000, #4631), p-p53^ser15^ (#9284), p-ERK1/2 (1:2000, #9106), p-CHK1^ser345^ and p-CHK2^ser19^ (#9931), Promega Corporation-Spain: p-JNK (#V7932), or Sigma-Aldrich: α-tubulin (1:10000, #TP026). Unless indicated, all antibodies were diluted 1:1000. Secondary antibodies conjugated with horseradish peroxidase were purchased from BioRad, and chemiluminescence detection was performed using ECL (Santa Cruz Biotechnology).

### Confocal microscopy

Tumor cells were seeded at a density of 3 × 10^5^ cells in 15 µm 8-well IBIDI plates and incubated overnight. Cells were then treated with compounds at their IC_50_ concentrations and incubated for 1 h. Subsequently, cells were stained with 10 μM of dihydroethidium (DHE, cytosolic O_2_^·-^ probe, Sigma-Aldrich) or with 5 μM of Mitosox (mitochondrial O_2_^·-^ probe, Sigma-Aldrich) for 15–30 min^79^ and then visualized on a confocal microscope (Confocal LSM 710 Zeiss, Biomedical Research Institute Alberto Sols, IIBm-CSIC-UAM, Madrid, Spain). The image analysis was realized using the software program Image J.

### Ratio GSH/GSSG

GSH and GSSG levels were determined in AGS and PANC1 cells after 3 and 24 h of treatment with the compounds (using H_2_O_2_ as a positive control) with the Glutathione Colorimetric Detection Kit (ThermoFisher) following the manufacturer’s instructions. Thirty minutes after adding the Colorimetric Detection Reagent and the Reaction Mixture, the absorbance was read at 405 nm. A standard curve was generated with each assay to interpolate the sample results. To measure GSSG, standards and samples must be diluted with the Sample Diluent containing 2-vinylpyridine.

### Clonogenic assay

For the clonogenic assay we used two different approaches. In Fig. S7b, PANC1 cells were treated with the metallodrugs for 1, 3 and 24 h (IC_50_ concentrations). Then, cells were cultured in a 6-well plate at a very low concentration (2 × 10^3^ cells per well) and incubated for 10 days. In Fig. S6b GC cells were cultured in a 6-well plate at a very low concentration (2 × 10^3^ cells per well), incubated overnight to ensure attachment and then treated with the IC_50_ and a suboptimal concentration close to the IC_25_ of the compounds. To evaluate the role of ROS in cell proliferation, we supplemented the growth medium with 0.5 mM NAC. After 10 days, the cells were fixed with 1% glutaraldehyde and stained with 0.1% crystal violet. The number of colonies was quantified directly and reflected as CFUs: Colony Forming Units.

### Indirect calorimetry analyses (PhenoMaster)

Indirect calorimetry analyses were carried out using a 16-chamber TSE PhenoMaster monitoring system (TSE Systems GmbH, Bad Homburg, Germany). Full access to food and water was continuously available, and intake was monitored using built-in devices located within each cage. Calorimetry measurements were carried out during a period of 72 h, according to animal weight, to exclude changes in body weight that would contribute to differences in energy expenditure measurements^80^. Twenty-four hours prior to introducing mice into the PhenoMaster monitoring system, 10 week-old C57Bl6 mice (Janvier, France) were injected intravenously (i.v.; tail vein injection) with 100 µl of I5 (concentrated stock in DMSO) diluted in physiological saline (0.9% NaCl) to 1.4 mg/kg or 100 µl of physiological saline (i.e., Sham Control). Mice were introduced into individual chambers and were on a 12 h light-dark cycle (lights on at 07:00am) during the course of the experiment, with a maintained room temperature of 22 ± 2°C. Oxygen consumption and CO2 release was measured. From these values, respiratory exchange ratio (RER) was determined as VCO2/VO2 and energy expenditure (EE) was calculated as = (3.185 + 1.232 x RER) x VO2. Locomotor activity was reported as the average light beam breaks (XT + YT) per min. XT and YT (T = total) were calculated from the sum of the ambulatory and fine movements. Water and food consumption (DRINK and FOOD, respectively, Fig. S8) were also monitored and recorded.

### Tumor xenograft models

Two in vivo approximations were used: (i) For pre-treatment in vivo experiments, 1 × 10^5^ PANC1 cells were treated with 20 μM of I5 or diluent control 24 h before trypsinization. PANC1 cells were then trypsinized and resuspended in RPMI culture medium with 40% Matrigel (no. 45354234, Corning® Matrigel® Basement Membrane Matrix, LDEV-free, Cultek, Spain) at a concentration of 1 × 10^5^ cells per 50 μL, and then 50 μL of the resuspended cells were subcutaneously injected into the right and left flanks of female 8 week-old NU-Foxn1nu nude mice (Janvier Labs, France). Tumor growth and volumes were monitored every 2–3 days for up to 4 weeks with a caliper, and tumor volumes (mm^3^) were computed using the formula: 0.5 × D12 × D2, where D1 and D2 are the width or the largest diameter and the length or the smallest diameter of a given tumor, respectively. At the end of the experiment, mice were sacrificed, and tumors extracted, photographed, and weighed. (ii) For xenograft in vivo treatment experiments, 1 × 10^6^ PANC1 cells resuspended in RPMI culture medium supplemented with 40% Matrigel (Corning®) were orthotopically injected in the pancreas of female NU-Foxn1nu nude mice (Janvier Labs, France). Approximately 2 months post injection, tumors were excised, cut into identical pieces of ~50 mm^3^ and implanted (with RPMI culture medium supplemented with 40% Matrigel® Corning®) subcutaneously into the left and right flanks of 15 8 week-old NU-Foxn1nu nude mice (Janvier, France). Three weeks later, tumors were measured to ensure volumes of 125–150 mm^3^, mice were weighed to calculate treatment concentrations per Kg, randomized into treatment groups (5 mice per group) and treatments were initiated for approximately three consecutive weeks. I5 stock (diluted in DMSO) was resuspended in physiological saline (0.9% NaCl) to a concentration of ~1.4 mg/Kg. Two routes of administration for I5 were tested: (1) via retro-orbital injection (r.o., 100 µl, 3 times/week) or (2) intraperitoneally (i.p., 100 µl, 3 times/week). Control mice were treated with 100 µl of physiological saline (0.9% NaCl). Tumor volumes were determined twice per week by caliper measurements throughout the course of the study as described above. At the indicated end point, mice were sacrificed, weighed, blood was collected in EDTA tubes (Aquisel, Cat no. 107545), urine was collected in 1.5 ml Eppendorf tubes, and tumors were excised, weighed, photographed, and fixed in 4% Paraformaldehyde (Alfa Aesar™).

Whole blood was analyzed with an Element HT5, Veterinary Hematology Analyzer (Scil animal care company GmbH, Madrid, Spain) and plasma was analyzed at the Analytical Laboratory of the Hospital Fundación Jiménez Díaz. Plasma-EDTA samples were examined for markers of renal (urea, BUN, and creatinine) and liver [bilirubin and GGT (γ-Glutamyltransferase)] injury. Urine was analyzed for protein, creatinine and glucose. Urea, creatinine, and glucose were detected by enzymatic assays, total protein by turbidimetry, and bilirubin and GGT via a colorimetric assay in the Cobas® Roche 701 module (a fully automated, high throughput photometric analyzer for a large array of quantitative and qualitative in vitro tests), following the manufacturer’s instructions.

### Ethics

All in vivo procedures in mice were conducted in accordance with protocols approved by the Use Committee for Animal Care from the Universidad Autónoma de Madrid (UAM) (Ref# CEI-25-587) and the Comunidad de Madrid (PROEX 294/19). For all in vivo experiments, mice were housed according to institutional guidelines and all experimental procedures were performed in compliance with the institutional guidelines for the welfare of experimental animals and in accordance with the guidelines for Ethical Conduct in the Care and Use of Animals as stated in The International Guiding Principles for Biomedical Research involving Animals, developed by the Council for International Organizations of Medical Sciences (CIOMS).

### Statistics and reproducibility

Statistical analyses were performed using GraphPad prism 8.0 Student’s 2-tailed *t*-test and one-way ANOVA with Dunnett post-test. Values of *P < 0.05 were considered significant.

### Reporting summary

Further information on research design is available in the Nature Portfolio Reporting Summary linked to this article.

### Supplementary information

Reporting Summary

Supplementary information

Description of Additional Supplementary Files

Supplementary Data

## Data Availability

All data supporting the findings of this study are available within the paper and its supplementary information. All the uncropped blots are collected in Fig. S11. The source data behind the graphs in the main and Supplementary Figures can be found in the Supplementary Data file.
