# Supplementary material for: Platinum iodido drugs show potential anti-tumor activity, affecting cancer cell metabolism and inducing ROS and senescence in gastrointestinal cancer cells
Source: Commun Biol. 2024 Mar 22;7:353. doi: 10.1038/s42003-024-06052-5 (PMC10959927; doi:10.1038/s42003-024-06052-5)
Supplement: Supplementary file 1 — Reporting Summary [file 42003_2024_6052_MOESM1_ESM.pdf]

nature portfolio

Corresponding author(s): Sánchez-Pérez I and Sainz, Jr. B

Last updated by author(s): Feb 23, 2024

Reporting Summary

Nature Portfolio wishes to improve the reproducibility of the work that we publish. This form provides structure for consistency and transparency in reporting. For further information on Nature Portfolio policies, see our Editorial Policies and the Editorial Policy Checklist.

Statistics

For all statistical analyses, confirm that the following items are present in the figure legend, table legend, main text, or Methods section.

n/a	Confirmed	
☐	☒	The exact sample size (*n*) for each experimental group/condition, given as a discrete number and unit of measurement
☐	☒	A statement on whether measurements were taken from distinct samples or whether the same sample was measured repeatedly
☐	☒	The statistical test(s) used AND whether they are one- or two-sided<br>*Only common tests should be described solely by name; describe more complex techniques in the Methods section.*
☒	☐	A description of all covariates tested
☒	☐	A description of any assumptions or corrections, such as tests of normality and adjustment for multiple comparisons
☐	☒	A full description of the statistical parameters including central tendency (e.g. means) or other basic estimates (e.g. regression coefficient) AND variation (e.g. standard deviation) or associated estimates of uncertainty (e.g. confidence intervals)
☐	☒	For null hypothesis testing, the test statistic (e.g. *F*, *t*, *r*) with confidence intervals, effect sizes, degrees of freedom and *P* value noted<br>*Give P values as exact values whenever suitable.*
☒	☐	For Bayesian analysis, information on the choice of priors and Markov chain Monte Carlo settings
☒	☐	For hierarchical and complex designs, identification of the appropriate level for tests and full reporting of outcomes
☒	☐	Estimates of effect sizes (e.g. Cohen's *d*, Pearson's *r*), indicating how they were calculated

Our web collection on statistics for biologists contains articles on many of the points above.

Software and code

Policy information about availability of computer code

Data collection	Invitrogen™ Attune™ NxT software, version 3.1.1 was used for cytometry data collection<br>BD FACSDivaTM software v.9.0 was used for FACS data collection<br>XF96 1.4.2 Software (Agilent) for Seahorse data collection<br>The program Zen2 (Blue Edition) was used to obtain images from the laser scanning confocal microscope Zeiss 710, and Inmunofluoresce microscope NiKON i90<br>Quantitative PCR Step One Software v2.3 Applied Biosystems.<br>Fusion Solo 6S Edge. Fusion-Capt Software. Vilber<br>TSE PhenoMaster monitoring system (TSE Systems GmbH, Bad Homburg, Germany)<br>Cobas® Roche 701 module<br>Element HT5, Veterinary Hematology Analyzer (Scil animal care company GmbH, Madrid, Spain)<br>VERSA max microplate reader molecular devices IIBM CSIC-UAM

Data analysis

For image preparation and analysis: Fiji package of ImageJ V2.0.0-rc-64/1.51s.
For flow cytometry image preparation and analysis: FlowJo 9.3 software (Tree Star Inc., Ashland, OR.)
For graphs preparation and analysis: Prism 8.0 GraphPad Software, Inc
For OCR and ECAR analsyses: XF96 1.4.2 Software (Agilent)
For cell viability Softmax Pro Molecular devices.
For Western Blot acquisition Fusion Software Vilber
For Immunofluorescence acquisition Nikon NIS elements
For PCR Step one software V.2.3 Applied Biosystems

For manuscripts utilizing custom algorithms or software that are central to the research but not yet described in published literature, software must be made available to editors and reviewers. We strongly encourage code deposition in a community repository (e.g. GitHub). See the Nature Portfolio guidelines for submitting code & software for further information.

Data

Policy information about availability of data

All manuscripts must include a data availability statement. This statement should provide the following information, where applicable:

- Accession codes, unique identifiers, or web links for publicly available datasets
- A description of any restrictions on data availability
- For clinical datasets or third party data, please ensure that the statement adheres to our policy

All data supporting the findings of this study are available within the paper and its supplementary information. All the uncropped blots are collected in Fig.S11.

Research involving human participants, their data, or biological material

Policy information about studies with human participants or human data. See also policy information about sex, gender (identity/presentation), and sexual orientation and race, ethnicity and racism.

Reporting on sex and gender

Use the terms sex (biological attribute) and gender (shaped by social and cultural circumstances) carefully in order to avoid confusing both terms. Indicate if findings apply to only one sex or gender; describe whether sex and gender were considered in study design; whether sex and/or gender was determined based on self-reporting or assigned and methods used.
Provide in the source data disaggregated sex and gender data, where this information has been collected, and if consent has been obtained for sharing of individual-level data; provide overall numbers in this Reporting Summary. Please state if this information has not been collected.
Report sex- and gender-based analyses where performed, justify reasons for lack of sex- and gender-based analysis.

Reporting on race, ethnicity, or other socially relevant groupings

Please specify the socially constructed or socially relevant categorization variable(s) used in your manuscript and explain why they were used. Please note that such variables should not be used as proxies for other socially constructed/relevant variables (for example, race or ethnicity should not be used as a proxy for socioeconomic status).
Provide clear definitions of the relevant terms used, how they were provided (by the participants/respondents, the researchers, or third parties), and the method(s) used to classify people into the different categories (e.g. self-report, census or administrative data, social media data, etc.)
Please provide details about how you controlled for confounding variables in your analyses.

Population characteristics

Describe the covariate-relevant population characteristics of the human research participants (e.g. age, genotypic information, past and current diagnosis and treatment categories). If you filled out the behavioural & social sciences study design questions and have nothing to add here, write "See above."

Recruitment

Describe how participants were recruited. Outline any potential self-selection bias or other biases that may be present and how these are likely to impact results.

Ethics oversight

Identify the organization(s) that approved the study protocol.

Note that full information on the approval of the study protocol must also be provided in the manuscript.

Field-specific reporting

Please select the one below that is the best fit for your research. If you are not sure, read the appropriate sections before making your selection.

☒ Life sciences ☐ Behavioural & social sciences ☐ Ecological, evolutionary & environmental sciences

For a reference copy of the document with all sections, see nature.com/documents/nr-reporting-summary-flat.pdf

Life sciences study design

All studies must disclose on these points even when the disclosure is negative.

Sample size

No statistical methods were used to predetermine the sample size. Sample sizes for experiments were estimated based on previous experience with a similar setup that showed significance. Published examples include (Sancho P, et al. MYC/PGC-1α Balance Determines the Metabolic Phenotype and Plasticity of Pancreatic Cancer Stem Cells. Cell Metab. 2015 Oct 6;22(4):590-605. PMID: 26365176; and Hermann PC et al. Multimodal Treatment Eliminates Cancer Stem Cells and Leads to Long-Term Survival in Primary Human Pancreatic Cancer Tissue

Xenografts. PLoS One. 2013 Jun 18;8(6):e66371. PMID: 23825539, N. Pajuelo-Lozano, et al. . XPA, XPC, and XPD Modulate Sensitivity in Gastric Cisplatin Resistance Cancer Cells. Front Pharmacol. 9 (2018) 1197. https://doi.org/10.3389/fphar.2018.01197, N. Pajuelo-Lozano, et al.Targeting MAD2 modulates stemness and tumorigenesis in human Gastric Cancer cell lines Theranostics 2020 Jul 25;10(21):9601-9618 doi: 10.7150/thno.49270. eCollection 2020.). Experiments involved mice were performed two to three times (as indicated) and 6-9 animals where analyzed for each experiments/ time point. Each study was designed to use the minimum number of mice required to obtain informative results (that is, quantitative data amenable to statistical analysis).

Data exclusions	No data were excluded
Replication	Experiments were repeated and experimental findings were reproducible. Specifically, we have included in the manuscript the following reproducibility statement "The number of biologically independent samples are indicated in the Figure legends. Repeated independent experiments per each panel with similar results are shown below. N=3
Randomization	For the in vivo experiments, mice were previously randomized into experimental groups. Cell line experimentation: For each experiment, the total amount of cells from one cell line required for all tested conditions were pooled and seeded randomly into different plates, pre-labeled with the treatment to be applied.
Blinding	No formal blinding was used. Tumor measurements were conducted by an independent technician who was unaware of the hypothesis or the conditions of the cells injected. Codification of the samples injected was maintained during cell injections. The analysis post mouse sacrifice was performed by an author who using the coded samples. Decoding was done post analysis. For the imaging experiments, including Confocal microscopy, where manual counting was required, samples were labeled with numbers in order to avoid preconceptions of the analyzing investigator. Automated software was used for FACS analysis, precluding bias of the investigator. If settings other than the default settings of a software was used, the parameters are specified in the respective methodology section. The remaining experiments were analyzed by western blotting or RTqPCR. In this case, codification was maintained during sample preparation. Decoding was done before loading the samples on the SDS-PAGE gel or loading the PCR plate, in order to ensure an adequate presentation of the results.

Reporting for specific materials, systems and methods

We require information from authors about some types of materials, experimental systems and methods used in many studies. Here, indicate whether each material, system or method listed is relevant to your study. If you are not sure if a list item applies to your research, read the appropriate section before selecting a response.

Materials & experimental systems

n/a	Involved in the study
☐	☒ Antibodies
☐	☒ Eukaryotic cell lines
☒	☐ Palaeontology and archaeology
☐	☒ Animals and other organisms
☒	☐ Clinical data
☒	☐ Dual use research of concern
☒	☐ Plants

Methods

n/a	Involved in the study
☒	☐ ChIP-seq
☐	☒ Flow cytometry
☒	☐ MRI-based neuroimaging

Antibodies

Antibodies used	WB=Western Blot; IF: Immunofluorescence<br>α-Mcl1, Rabbit Policlonal 1:1000 WB, Santa Cruz Technology (sc-819)<br>Proapoptosis Bcl-2 Kit: α-BIM, α-BID, α-BAX, Rabbit Monoclonal 1:1000 WB, Cell Signaling Technology (#9942)<br>α-p-p38 (Thr180/Tyr182), Rabbit Monoclonal 1:2000 WB, Cell Signaling Technology (#4631)<br>α-p-p38 (ser15), Rabbit Monoclonal 1:1000 WB, Cell Signaling Technology (#9284)<br>α-p-ERK1/2 (Thr202/Tyr204), Mouse Monoclonal 1:2000 WB, Cell Signaling Technology (#9106)<br>Phospho-Chk1/2 Antibody Sampler Kit: α-p-CHK1 (ser345), α-p-CHK2 (ser19), Rabbit Monoclonal 1:1000 WB, Cell Signaling Technology (#9931)<br>α-p-JNK1/2, Rabbit Policlonal 1:1000 WB, Promega Corporation-Spain (#V7932)<br>α-tubulin, Mouse Monoclonal 1:10000 WB, Sigma-Aldrich (#T9026)<br>α-Histone H2AX, Rabbit Policlonal 1:100 IF, Cell Signaling Technology (#2595)<br>α-53BP1, Rabbit Policlonal 1:100 IF, Cell Signaling Technology (#4937)<br>Secondary Antibodies:<br>α-rabbit-HRP, Donkey 1:5,000 WB Amersham (Cat no. NA9340-1ML)<br>Anti-rabbit IgG (H+L), F(ab') Fragment (Alexa Fluor 488 Conjugate)<br>α-Mouse IgGs-HRP Goat 1:5000 IHC DAKO (Cat no. P0447)
Validation	All antibodies used are commercially available and validated by the manufacturers, as indicated on the respective web sites of each commercial vendor. Please refer to the commercial website of each antibody for more details.

Eukaryotic cell lines

Policy information about cell lines and Sex and Gender in Research

Cell line source(s)	ATCC, DSMZ
Authentication	Cell lines were authenticated by microsatellite analysis in the "Servicio de Genómica" (IIBm-CSIC-UAM) prior to use.
Mycoplasma contamination	Mycoplasma contamination tests are frequently run (each month) in our laboratory via "Servicio de Cultivos Celulares" (IIBm-CSIC-UAM)
Commonly misidentified lines (See ICLAC register)	No commonly misidentified cell lines were used in the study.

Animals and other research organisms

Policy information about studies involving animals; ARRIVE guidelines recommended for reporting animal research, and Sex and Gender in Research

Laboratory animals	Female 8-week-old NU-Foxn1nu nude mice (Envigo, Spain) . Mice were housed according to the following guidelines: a 12 h light/12 h dark cycle, with no access during the dark cycle; temperatures of 65-75°F (~18-23°C) with 40-60% humidity; a standard diet with fat content ranging from 4% to 11; sterilized water was accessible at all times; for handling, mice were manipulated gently and as little as possible; noises, vibrations and odors were minimized to prevent stress and decreased breeding performance; and enrichment was always used per the facility's guidelines to help alleviate stress and improve breeding. These details are also included in the article.
Wild animals	The study did not involve wild animals.
Reporting on sex	Female mice were used for all in vivo studies as females can be grouped easily, while male mice cannot be grouped unless they originate from the same litters. As such and to avoid isolating a male mouse, which goes against standard animal facility protocols, female mice were used.
Field-collected samples	The study did not involve samples collected from field.
Ethics oversight	Mice were housed according to institutional guidelines and all experimental procedures were performed in compliance with the institutional guidelines for the welfare of experimental animals approved by the Universidad Autónoma de Madrid Ethics Committee (CEI 60-1057-A068 and CEI 103-1958-A337) and La Comunidad de Madrid (PROEX 335/14 and PROEX 294/19) and in accordance with the guidelines for Ethical Conduct in the Care and Use of Animals as stated in The International Guiding Principles for Biomedical Research involving Animals, developed by the Council for International Organizations of Medical Sciences (CIOMS).

Note that full information on the approval of the study protocol must also be provided in the manuscript.

Plants

Seed stocks	*Report on the source of all seed stocks or other plant material used. If applicable, state the seed stock centre and catalogue number. If plant specimens were collected from the field, describe the collection location, date and sampling procedures.*
Novel plant genotypes	*Describe the methods by which all novel plant genotypes were produced. This includes those generated by transgenic approaches, gene editing, chemical/radiation-based mutagenesis and hybridization. For transgenic lines, describe the transformation method, the number of independent lines analyzed and the generation upon which experiments were performed. For gene-edited lines, describe the editor used, the endogenous sequence targeted for editing, the targeting guide RNA sequence (if applicable) and how the editor was applied.*
Authentication	*Describe any authentication procedures for each seed stock used or novel genotype generated. Describe any experiments used to assess the effect of a mutation and, where applicable, how potential secondary effects (e.g. second site T-DNA insertions, mosiacism, off-target gene editing) were examined.*

Flow Cytometry

Plots

Confirm that:

☒ The axis labels state the marker and fluorochrome used (e.g. CD4-FITC).

☒ The axis scales are clearly visible. Include numbers along axes only for bottom left plot of group (a 'group' is an analysis of identical markers).

☒ All plots are contour plots with outliers or pseudocolor plots.

☒ A numerical value for number of cells or percentage (with statistics) is provided.

Methodology

Sample preparation	Cells were trypsinzed, blocked with Flebogamma and resuspended in Flow buffer [1X PBS; 3% FBS (v/v); 3mM EDTA (v/v)] with or without the appropriate diluted fluorescently-tagged antibody against the antigen of choice or with an IgG control.
Instrument	Invitrogen™ Attune™ NxT (cytometry), FACS Vantage SE Flow Cytometer (sorting)
Software	Invitrogen™ Attune™ NxT software, version 3.1.1 was used for cytometry data collection, BD FACSDiVa software was used for sorting data collection, and FlowJo 9.3 software (Tree Star Inc., Ashland, OR) was used for flow cytometry image preparation and analysis.
Cell population abundance	Purity is typically between 80-90% when confirmed using the Invitrogen™ Attune™ NxT
Gating strategy	Generally, cells were first gated on FSC-Area/FSC-Height to remove aggregates. Dead cells were removed by gating in DAPI-negative cells versus FSC-Area. Debris free, live, single cells were gated using FSC-Area and SSC-Area. Antigen or probe gating was performed on the live, single, debris free cell population. Gates were determined based on negative controls, unstained controls or IgG controls. The gating strategy used in all the Flow Cytometry experiments is reflected in Fig.S10

☒ Tick this box to confirm that a figure exemplifying the gating strategy is provided in the Supplementary Information.
