# Supplementary material for: Platinum iodido drugs show potential anti-tumor activity, affecting cancer cell metabolism and inducing ROS and senescence in gastrointestinal cancer cells
Source: Commun Biol. 2024 Mar 22;7:353. doi: 10.1038/s42003-024-06052-5 (PMC10959927; doi:10.1038/s42003-024-06052-5)
Supplement: Supplementary file 2 — Supplementary information [file 42003_2024_6052_MOESM2_ESM.pdf]

SUPPLEMENTARY INFORMATION

- Supplementary Figures S1-S11
- Supplementary Figure S1-S11 Legends
- Supplementary Table S1-S2

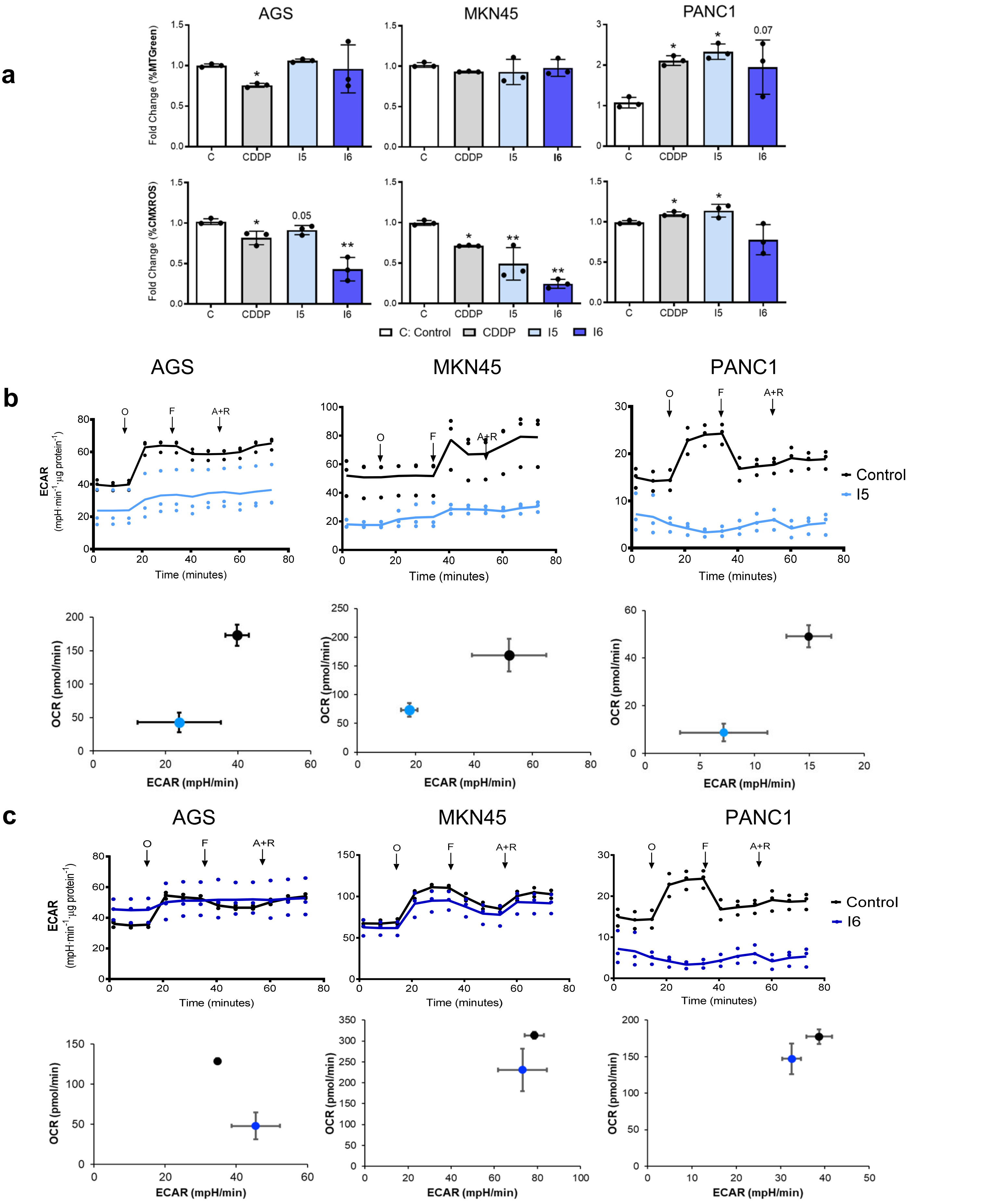

Figure S1. I5 and I6 affect gastric and pancreatic tumor cells mitochondrial parameters and metabolic phenotypes. a Mean fold-change ± SD in the mitochondrial mass probe MT Green (upper panel) and ΔΨm probe CMX-ROS (bottom panel) in AGS, MKN45 and PANC1 cells treated with CDDP, I5 and I6 IC_{50} doses (see Methods), 24 h. * $p < 0.05$, ** $p < 0.01$, as determined by unpaired two-sided Student's t-test, compared to untreated (C: Control) set as 1.0. **b-c)** Upper panels: Representative plots showing mean ± SD of extracellular acidification rate (ECAR), for untreated (Control) and I5- and I6-treated AGS, MKN45 and PANC1 cells (according to IC_{50} doses, 24 h), normalized to total protein using a BCA kit (measured as BCA absorbance). Continuous ECAR values (mpH/min/µg protein) are shown. Bottom panels: Representative energy maps for AGS, MKN45 and PANC1 cells treated with I5 and I6. Normalized ECAR and OCR data were plotted to reveal metabolic profile alterations for each cell line.

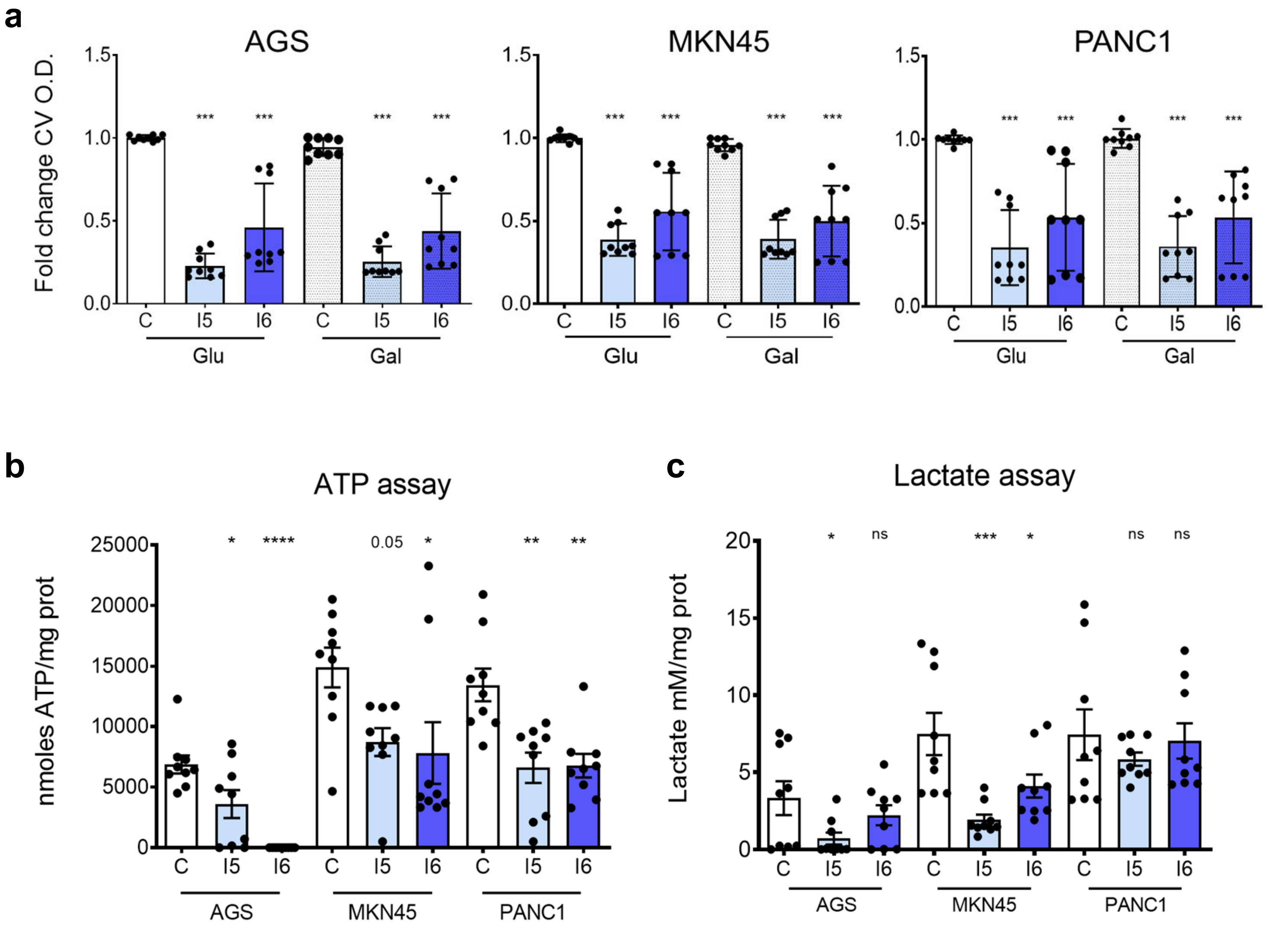

Figure S2. **I5 and I6 are metabolic inhibitors**. **a** Mean fold change ± SD in crystal violet optical density (CV O.D.) in AGS, MNK45 or PANC1 cells treated with I5 or I6 (IC50 concentrations, 24 h) and then cultured for 24 h in Glu (Glucose: OXPHOS-independent conditions) or Gal (Galactose: dependent conditions) (*** $p < 0.001$, as determined by unpaired Student's t test) compared to untreated (C) set as 1.0 (n=3). **b** ATP nmoles/mg protein (prot.) ± SEM determined in lysate pellets from untreated (C) and I5- or I6-treated AGS, MKN45 or PANC1 cells (IC50 concentrations, 24 h, n=3). (* $p < 0.05$, ** $p < 0.01$, **** $p < 0.0001$, as determined by unpaired Student's t test). Data were normalized to total protein using a BCA assay. **c** Lactate mM/mg protein (prot.) ± SEM determined in supernatant from AGS, MKN45 or PANC1 cells from control (C) and I5- or I6-treated AGS, MKN45 or PANC1 cells (IC50 concentrations, 24 h, n=3). (* $p < 0.05$, ** $p < 0.01$, *** $p < 0.001$, **** $p < 0.0001$, ns = not significant, as determined by unpaired Student's t test). Data were normalized to total protein using a BCA assay.

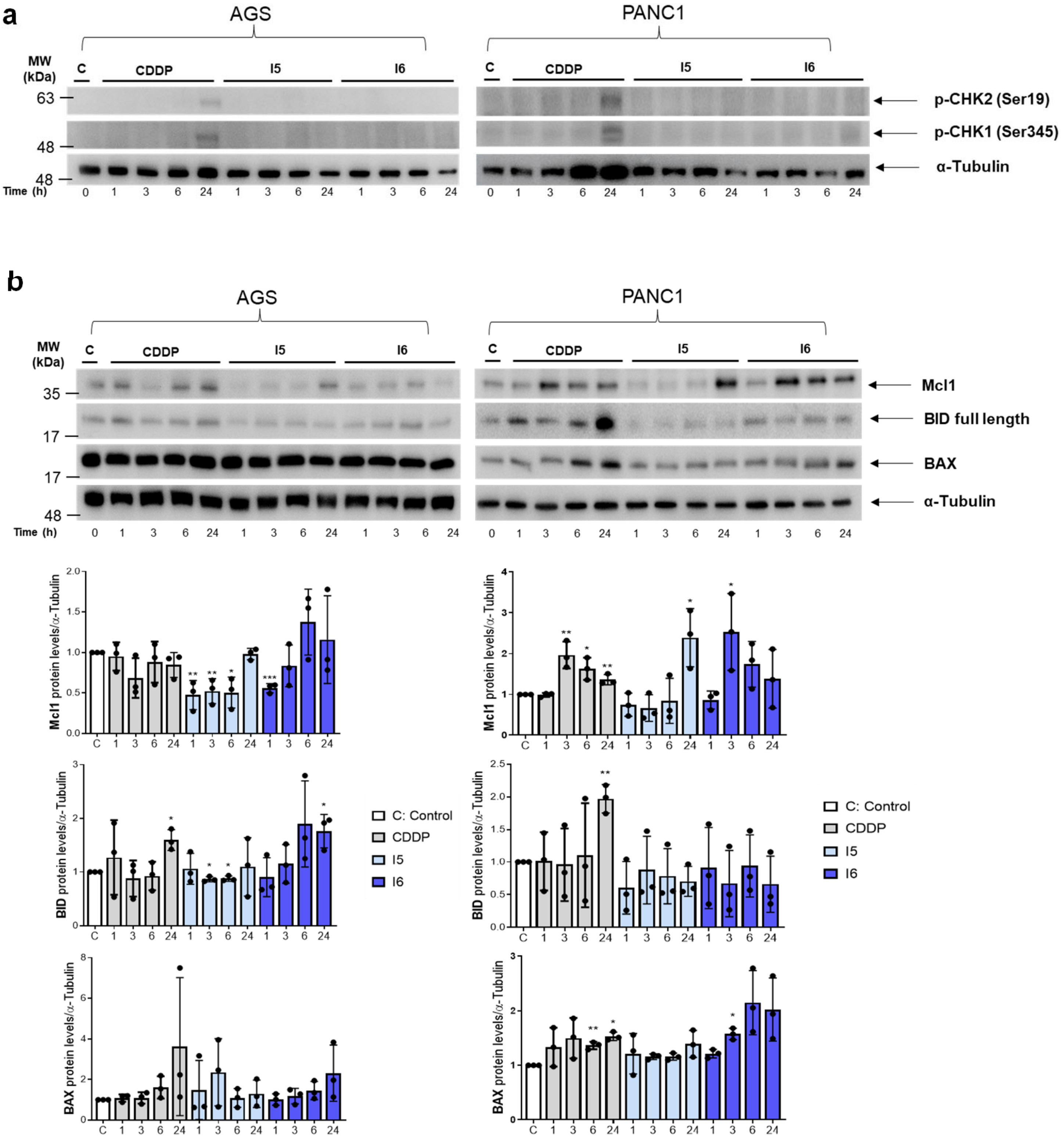

Figure S3. Western Blot analysis of apoptosis-associated proteins in AGS and PANC1 cells. Cells were treated with IC_{50} concentrations of CDDP, I5 or I6 (see Methods) at different times (1, 3, 6 or 24 h). **a** Representative western blots of DNA Damage Response (DDR) proteins: p-CHK1^{ser345}, p-CHK2^{ser19}. **b** Representative western blots of Bcl-2 protein family members: Mcl1, BID and BAX. α-Tubulin was used as endogenous control for all blots. Lower: Mean ± SD of the densitometry of bands for each indicated protein at indicated different times in n=3 separate experiments, determined using ImageJ (Area under the peak method) and normalized to α-Tubulin. The statistical significance was evaluated with Student's 2-tailed t-test (*p<0.05, **p<0.01, *** p < 0.001) compared to the untreated cells (C: Control), set as 1.0.

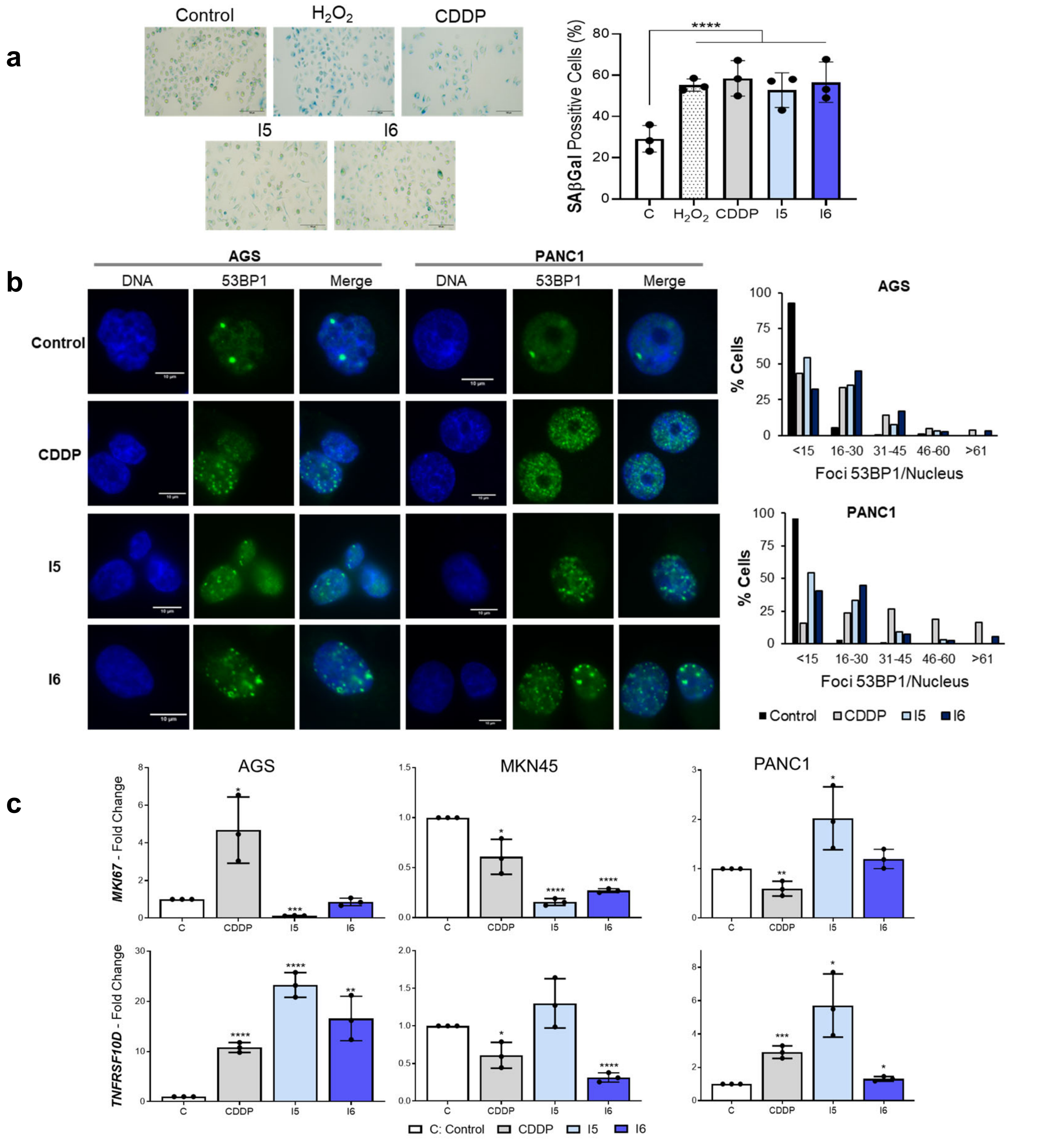

Figure S4. **I5 and I6 induce cellular senescence in GI cancer cells. a** The percentage of senescent cells was measured quantifying the SAβGal-positive staining with a histochemical kit (see Methods). AGS cells were treated for 3 h with the IC_{50} concentrations of CDDP, I5 or I6 (and H_2O_2 as positive control, see Methods) and then incubated 24 h with complete medium. Then, cells were fixed and stained. Left: Representative images of each condition were taken (Scale bar represents 100 µm). Right: Mean percentage of SAβGal positive cells ± SD. Statistical significance was evaluated with Student's 2-tailed t-test (****$p<0.0001$) compared to untreated (C), n=3. **b** 53BP1 expression is increased in GI cells stimulated with the platinum complexes. AGS and PANC1 cells were treated with the IC_{50} concentrations of CDDP, I5 or I6 for 3 h. 53BP1 foci (green fluorescence) were detected by immunofluorescence using DAPI to stain nuclear DNA (blue fluorescence). Left: Representative images of each condition. Scale bar represents 10 µm. Right: Mean percentage of nucleus within less than 15, between 16 and 30, between 31 and 45, between 46 and 60 and more than 60 53BP1 foci per nuclei for each condition. Data represent the mean values obtained in three experiments performed in duplicate. **c** RNA was isolated from AGS, MKN45 and PANC1 cell lines stimulated with a 24 h IC_{50}-treatment of the complexes. Shown are the mean fold-change ± SD of *MKI67* and *TNFRSF10D* mRNA expression quantified by RT-qPCR and normalized with *GAPDH*. Statistical significance was evaluated with Student's 2-tailed t-test (*$p<0.05$, **$p<0.01$, ***$p<0.001$, ****$p<0.0001$) compared to untreated (C) set as 1.0, n=3.

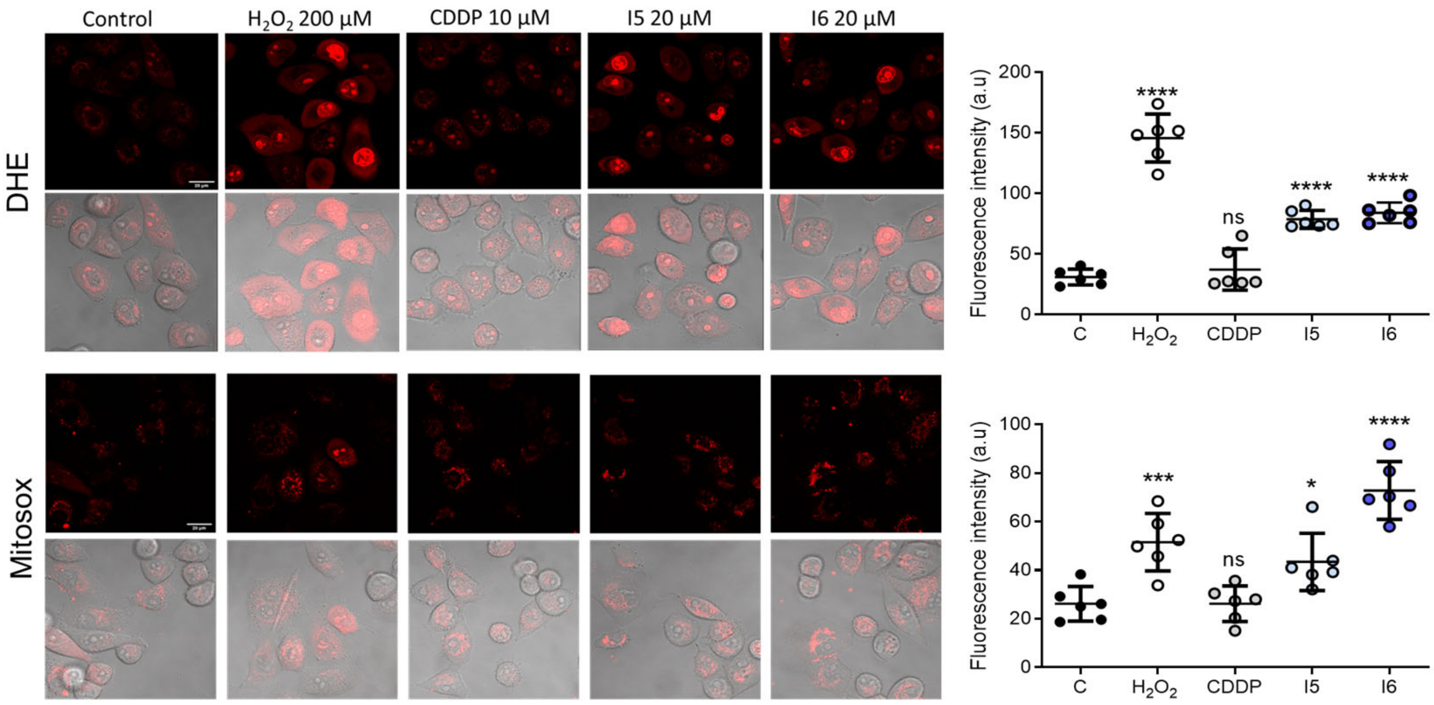

Figure S5. **I5 and I6 are ROS producers in PANC1 cells**. Cells were treated with the indicated compounds for 1 h followed by 30 min of incubation with the indicated probes. ROS levels were quantified by confocal microscopy using DHE and MitoSox as $O_2^{\bullet-}$ fluorescence indicator, in cytosol and mitochondria, respectively. Left: Representative images of each condition were taken. Scale bar represents 20 µm. Right: Mean fluorescence Intensity (per cell) ± SD was quantified and depicted in the graph. Graphs show a significant increase in the amount of ROS after the treatments: H_2O_2 (200 µM, white circles), CDDP (10 µM, grey circles), I5 (20 µM, light blue circles) and I6 (20 µM, dark blue circles). Statistical significance was evaluated with Student's 2-tailed t-test (ns= no significant, *p<0.05, **p<0.01, ***p<0.001) compared to the untreated cells (C: Control, black circles). The experiment was performed three independent times with duplicates.

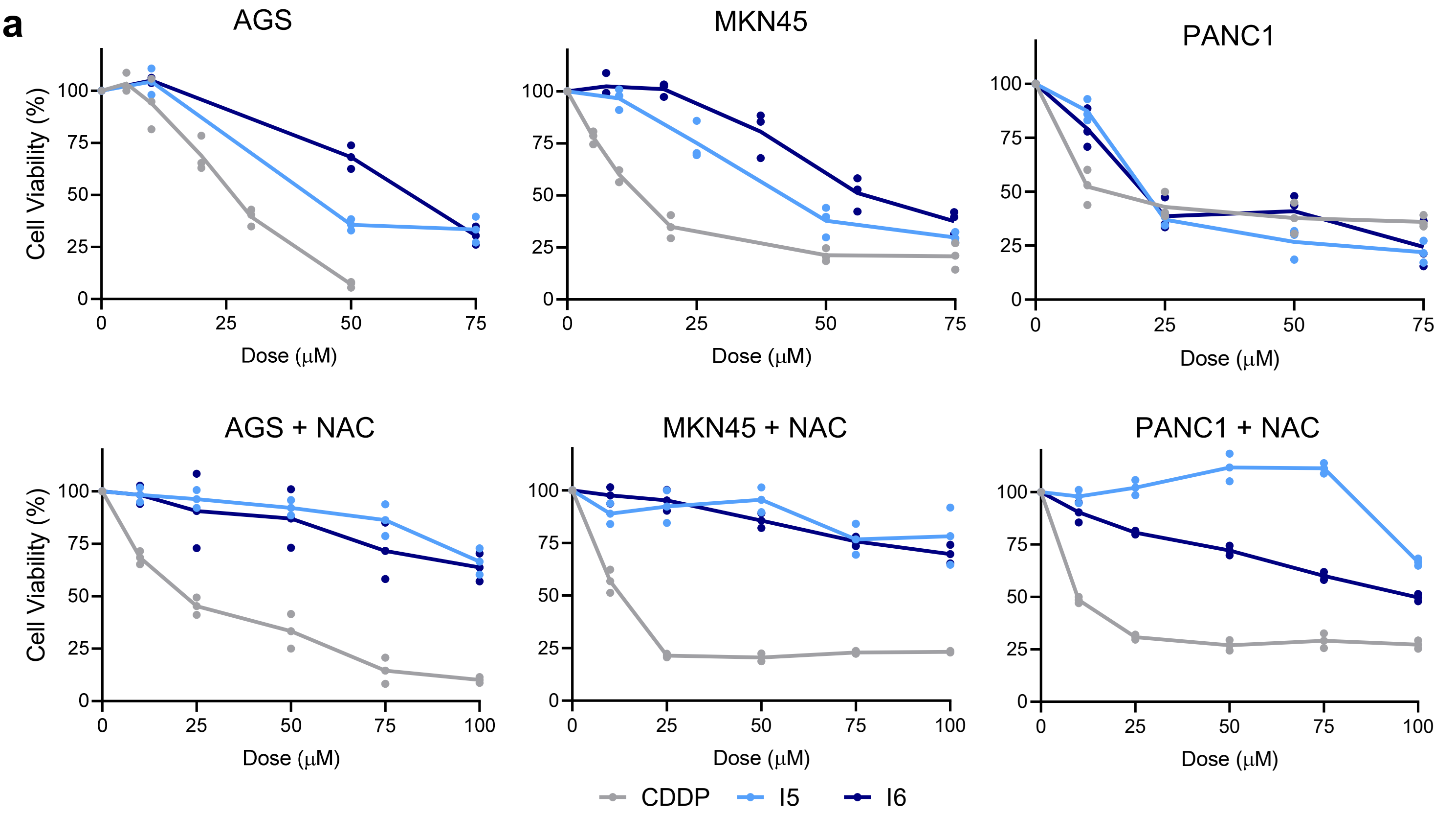

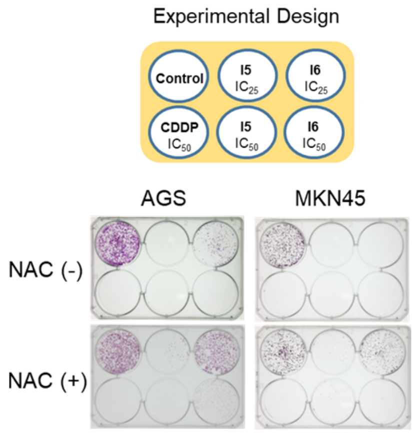

	AGS	AGS + NAC	MKN45	MKN45 + NAC
Control	638 ± 14	766 ± 114	724 ± 46	760 ± 107
CDDP (IC_{50})	0	0.7 ± 0.6	1 ± 1	1 ± 1.4
I5 (IC_{25})	0	106 ± 46	0	99 ± 6
I5 (IC_{50})	0	2.3 ± 2.5	0	52 ± 21
I6 (IC_{25})	268 ± 63	613 ± 89	1 ± 1.7	526 ± 93
I6 (IC_{50})	0	329 ± 152	0	46 ± 2

Figure S6. **I5 and I6 modulate cell viability and proliferation via oxidative damage**. **a** Effects of the complexes on cell viability in AGS (left panel), MKN45 (central panel) and PANC1 (right panel) cells, in the absence (top) or presence (bottom) of N-Acetylcysteine (NAC) 0.5 mM. Cells were treated with increasing concentrations (0-100 μM) of CDDP (grey), I5 (light blue) and I6 (dark blue). A protective effect is observed after the treatment with the iodido complexes in cells treated with NAC. Graphs show the mean percentage of viable cells after 48 h treatment, quantified by the CV assay, detailed in Methods. (n=3 independent experiments, performed in quadruplicate). IC_{50} were calculated with a nonlinear regression to fit the data to the log (inhibitor) versus response (variable slope). **b** Left: Experimental design of the Colony Forming Unit (CFU) assay used to determine cell proliferation. AGS and MKN45 cells were treated (for 10 days) with CDDP-IC_{50} or with the IC_{25} or IC_{50} concentrations of I5 or I6, in the presence or absence of N-Acetylcysteine (NAC) 0.5 mM. Representative images were taken. Right: Table summarizing the mean values ± SD of colonies (CFUs). Metallocomplexes had a marked effect on cell proliferation, but in those conditions with NAC, we observed an increase in the number of CFUs after the treatment with the iodido complexes. n=3.

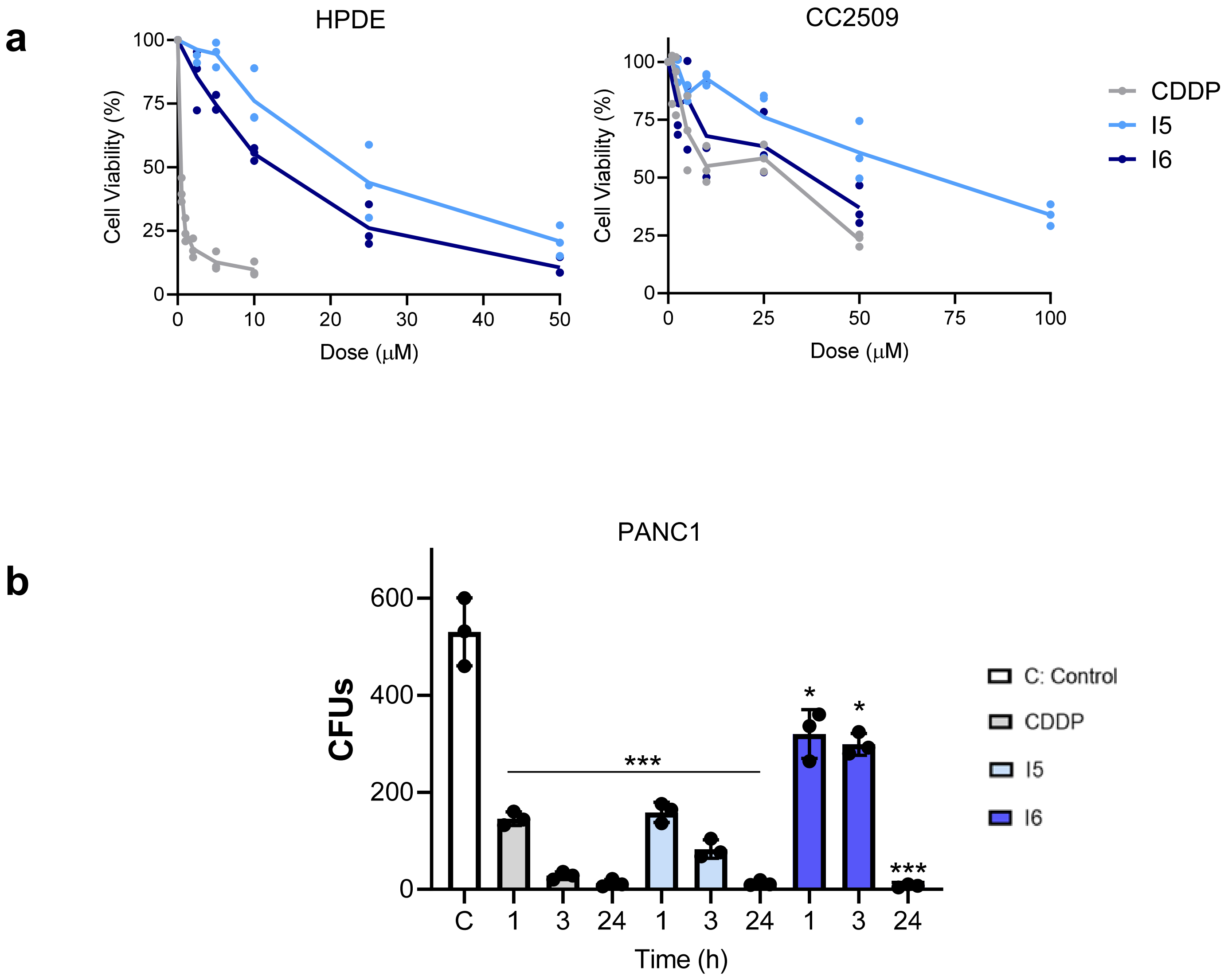

Figure S7. a I5 and I6 are less cytotoxic than CDDP in non-cancer cell lines. Effects of the complexes on cell viability in HPDE (left panel), and CC2509 (right panel) cells. Cells were treated with increasing concentrations of CDDP (grey), I5 (light blue) and I6 (dark blue). Graphs show the mean percentage of viable cells after 48 h treatment, quantified by the CV assay (detailed in Methods). (n=3 independent experiments, performed in quadruplicate). IC_{50} were calculated with a nonlinear regression to fit the data to the log (inhibitor) versus response (variable slope). **b I5 reduces in vitro clonogenicity.** Colony Forming Unit (CFU) assay was used to determine cell clonogenicity. PANC1 cells were treated 1, 3 and 24 h with the IC50 concentration of CDDP, I5 or I6, and colonies formed were stained with crystal violet and quantified 10 days later. Statistical significance was evaluated with Student's 2-tailed t-test (*p<0.05, ***p<0.001). N=3.

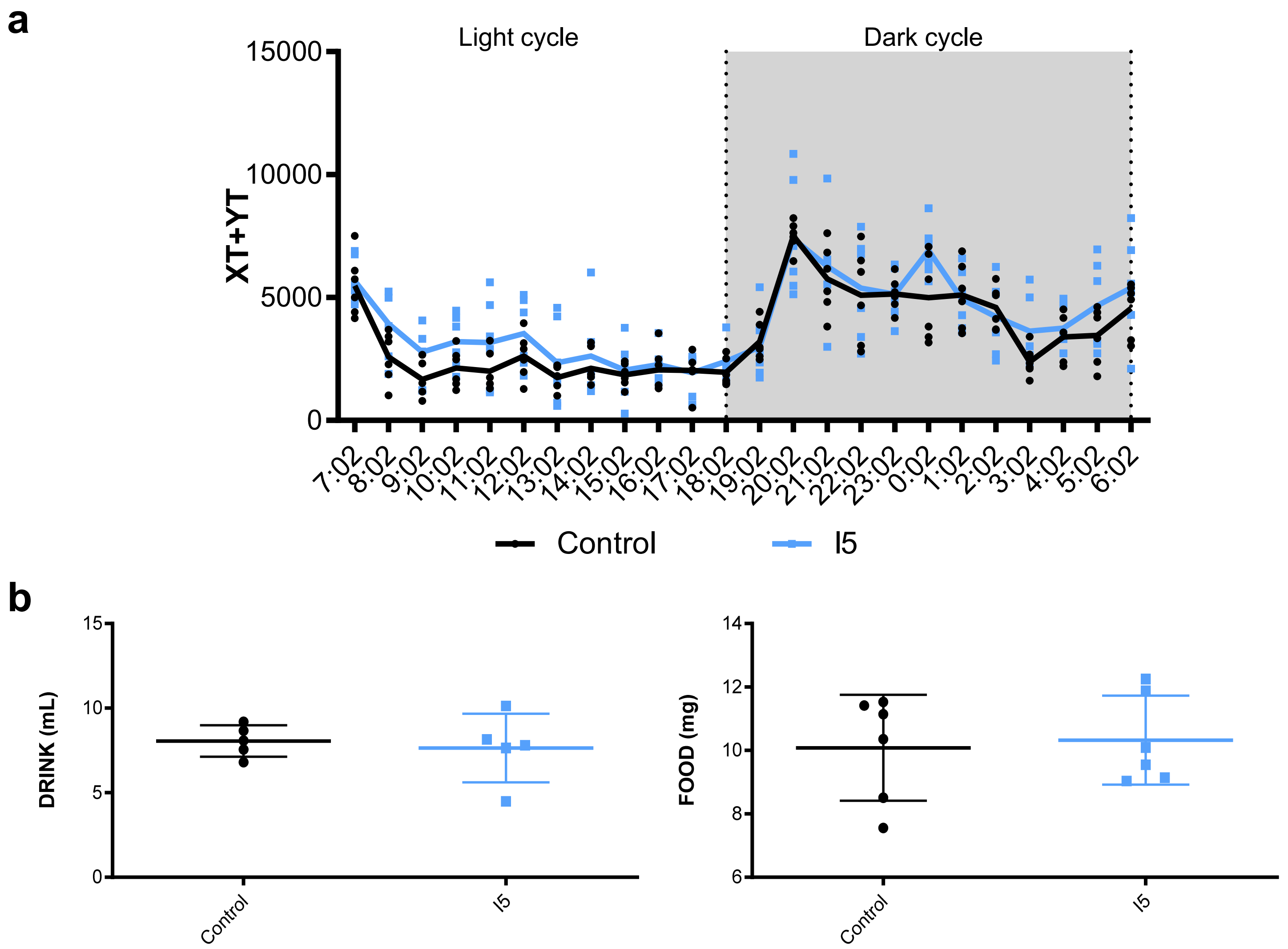

Figure S8. Indirect calorimetry analyses of mice treated with I5. a Locomotor activity was determined as the average light beam breaks (XT+YT) per min. XT and YT (T = total) were calculated from the sum of the ambulatory and fine movements. Shown are the mean XT+XY ± SD for mice treated intravenously with I5 (1.4 mg/Kg) or physiological saline (i.e., Control) as a function of time (24 hours). **b** Water (Drink) and food intake at the conclusion of the experiment. Shown are the mean DRINK or FOOF ± SD for mice treated intravenously with I5 (1.4 mg/Kg) or physiological saline (i.e., Control).

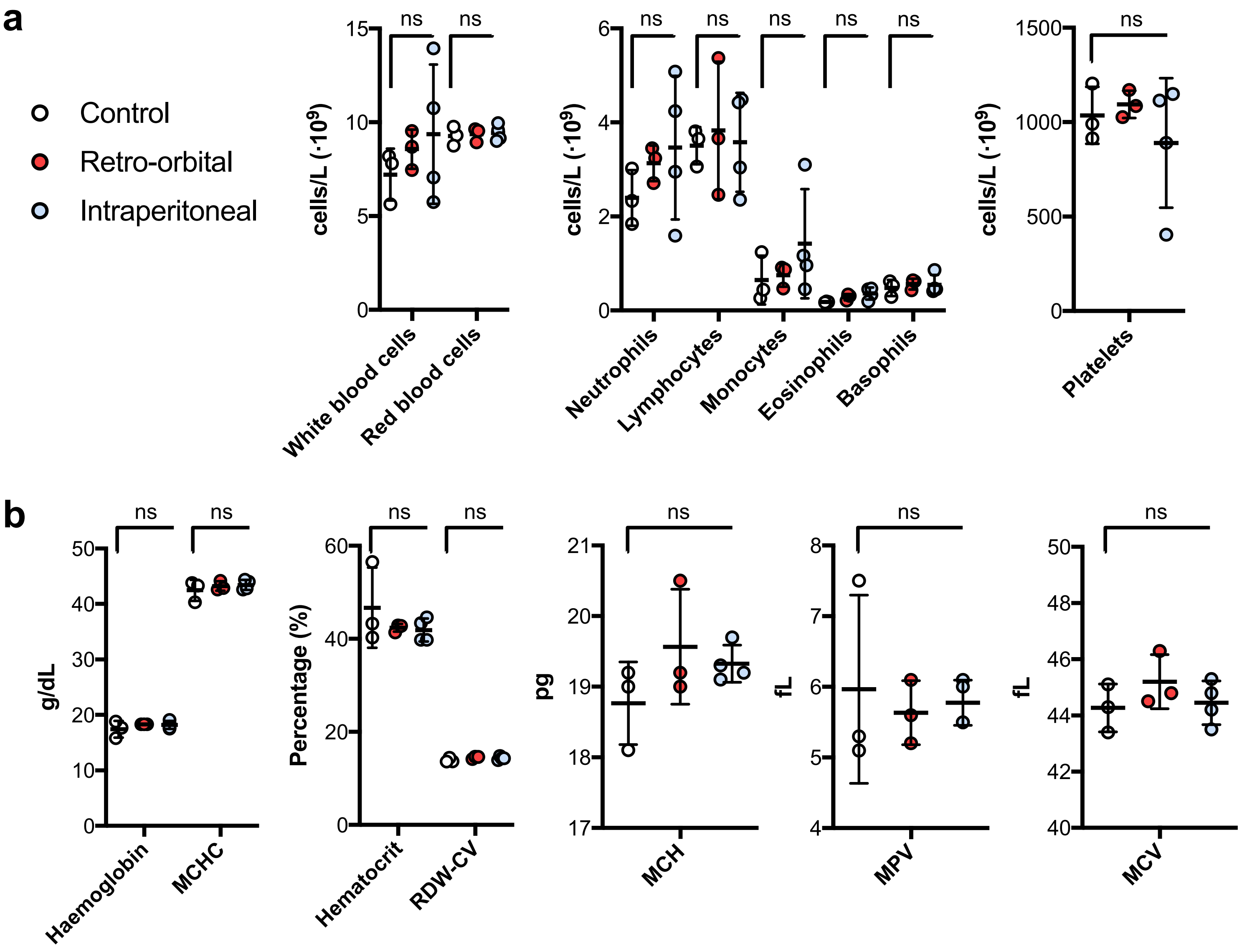

Figure S9. a-b Average values ± SD of indicated hematocrit parameters determined from blood of mice extracted on d17 post treatment with diluent Control or I5 (1.4mg/kg, r.o or i.p.). No significant differences were found, as determined by one-way ANOVA with Dunnett post-test, compared to diluent-treated (Control). n=3-4 mice/condition. MCHC = Mean Corpuscular Hemoglobin Concentration; RDW-CV = Red Blood Cell Distribution Width; MCH = Mean Corpuscular Hemoglobin; MPV = Mean Platelet Volume; MCV = Mean corpuscular volume; ns = not-significant.

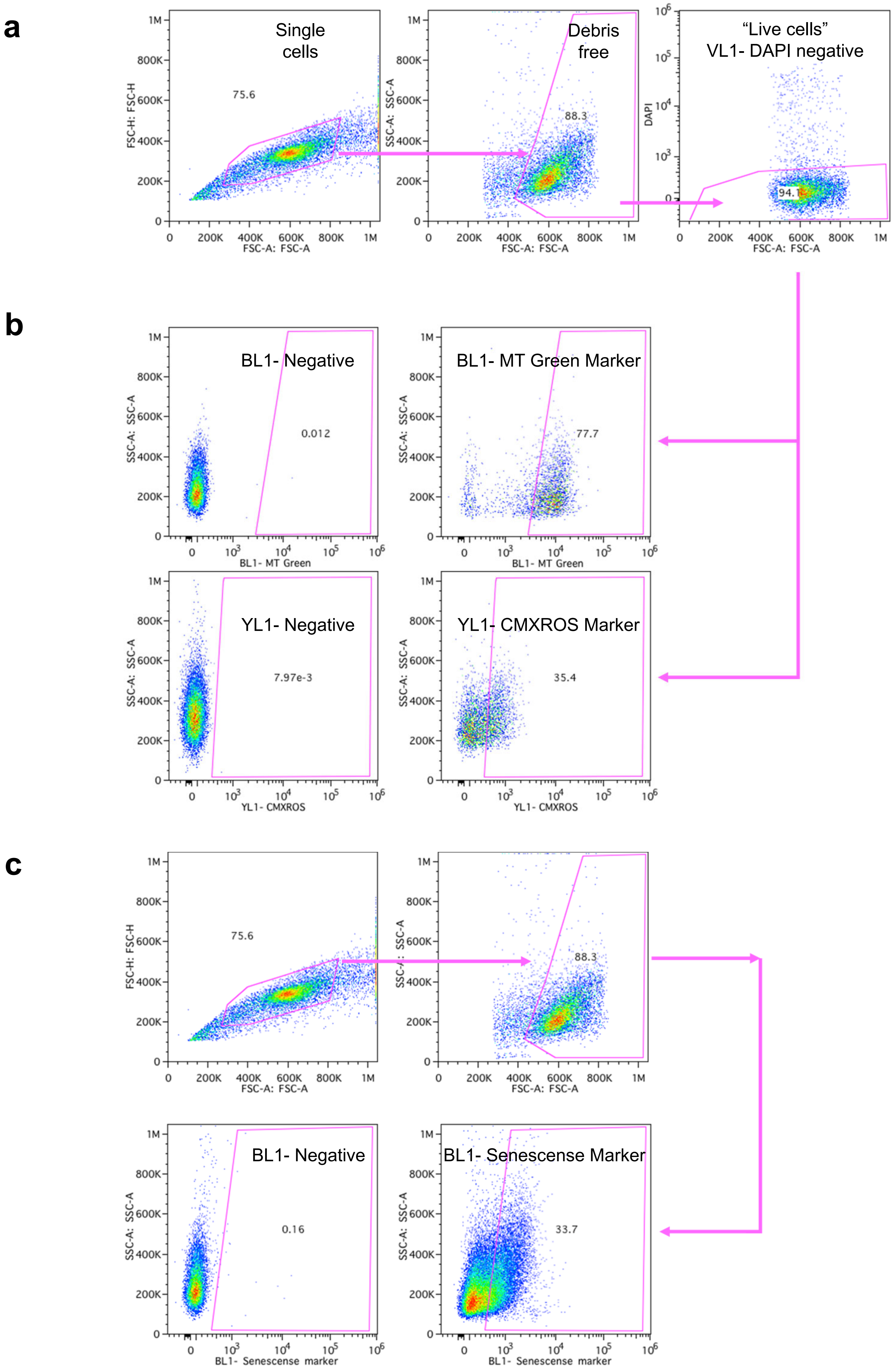

Figure S10. Flow cytometry gating strategies: a Initial gating strategy to achieve live cell population (one example showing initial gating strategies to acquire a single cell, debris free and DAPI-free ("live cell") population, applied to all analyses). **b** Gating strategies for different probes within "live cells". **c** Gating strategy for senescence probe (from fixed cells). Indicated below are the specific markers or probes for which the indicated gating strategy was applied. Additional details can be found in the Methods section.

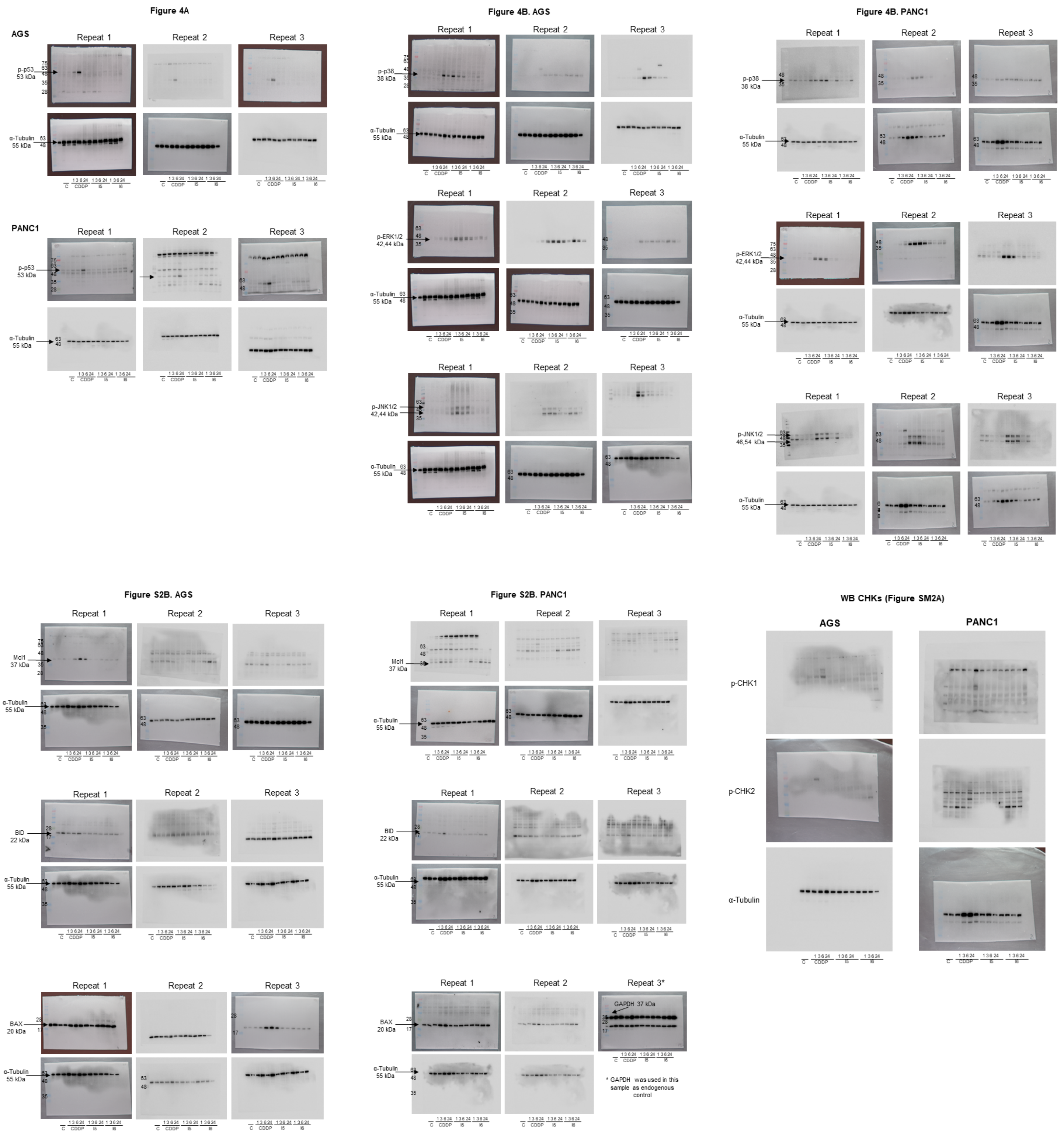

Figure S11. Uncropped Western Blotting Images.

Table S1. Biochemistry parameters in blood test in the three different experimental groups: C: Control (N=3), RO: Retro-Orbital (N=3) and IP: Intra-Peritoneal (N=4).

	Urea (mg/dL)	BUN (mg/dL)	Serum Creatinine (mg/dL)	Total Bilirubin (mg/dL)	GGT (UI/L)
**C-01**	41	19	0.18	<1.2	<3
**C-02**	38	18	0.18	<0.15	<3
**C-03**	39	18	0.18	<0.15	<3
**RO-01**	40	19	0.2	<0.15	<3
**RO-02**	35	16	0.21	<0.21	<3
**RO-03**	34	16	0.17	<0.15	<3
**IP-01**	40	19	0.2	<0.15	<3
**IP-02**	39	18	0.17	<0.15	<3
**IP-03**	38	18	0.22	<1.2	<3
**IP-04**	35	16	0.17	<1.2	<3

Table S2. Biochemistry parameters in urine test in the three different experimental groups: C: Control (N=3), RO: Retro-Orbital (N=3) and IP: Intra-Peritoneal (N=4).

	Protein in Urine (mg/dL)	Creatinine in Urine (mg/dL)	Protein/Creatinine in Urine	Glucose in Urine (mg/dL)
**C-01**	314	59	10744	36
**C-02**	106	82	12927	30
**C-03**	168	130	25840	40
**RO-01**	59	56	10536	30
**RO-02**	263	57	9221	30
**RO-03**	100	61	16383	30
**IP-01**	80	ND	ND	40
**IP-02**	52	59	8814	20
