# Supplementary material for: Platinum iodido drugs show potential anti-tumor activity, affecting cancer cell metabolism and inducing ROS and senescence in gastrointestinal cancer cells
Source: Commun Biol. 2024 Mar 22;7:353. doi: 10.1038/s42003-024-06052-5 (PMC10959927; doi:10.1038/s42003-024-06052-5)
Supplement: Supplementary file 3 — Description of Additional Supplementary Files [file 42003_2024_6052_MOESM3_ESM.pdf]

Description of Additional Supplementary Files

File name: Supplementary Data

Description: The source data behind the graphs in Figures 1-6, and Supplementary Figures 1-9.
